# Discovery and characterization of single nucleotide polymorphisms in two anadromous alosine fishes of conservation concern

**DOI:** 10.1002/ece3.3215

**Published:** 2017-07-18

**Authors:** Diana S. Baetscher, Daniel J. Hasselman, Kerry Reid, Eric P. Palkovacs, John Carlos Garza

**Affiliations:** ^1^ Department of Ocean Sciences University of California Santa Cruz CA USA; ^2^ Southwest Fisheries Science Center National Marine Fisheries Service Santa Cruz CA USA; ^3^ Department of Ecology and Evolutionary Biology University of California Santa Cruz CA USA; ^4^ Present address: Columbia River Inter‐Tribal Fish Commission Hagerman ID USA

**Keywords:** SNPs, alewife, blueback herring, anadromous, conservation, fisheries management

## Abstract

Freshwater habitat alteration and marine fisheries can affect anadromous fish species, and populations fluctuating in size elicit conservation concern and coordinated management. We describe the development and characterization of two sets of 96 single nucleotide polymorphism (SNP) assays for two species of anadromous alosine fishes, alewife and blueback herring (collectively known as river herring), that are native to the Atlantic coast of North America. We used data from high‐throughput DNA sequencing to discover SNPs and then developed molecular genetic assays for genotyping sets of 96 individual loci in each species. The two sets of assays were validated with multiple populations that encompass both the geographic range and the known regional genetic stocks of both species. The SNP panels developed herein accurately resolved the genetic stock structure for alewife and blueback herring that was previously identified using microsatellites and assigned individuals to regional stock of origin with high accuracy. These genetic markers, which generate data that are easily shared and combined, will greatly facilitate ongoing conservation and management of river herring including genetic assignment of marine caught individuals to stock of origin.

## 1 INTRODUCTION

Genetic data are routinely used to inform ecological investigation and formulate conservation and management plans for fish and wildlife. Elucidation of population structure and patterns of connectivity are often the first steps in the use of genetic data to understand a species’ biology. In addition, common applications for such data include reconstructing pedigree relationships, inferring historical demography, individual identification for mark/recapture type analyses, and evaluating patterns of natural selection and the identification of individuals to population of origin (Morin, Luikart, & Wayne, 2004; Narum et al., 2008).

Alewife (*Alosa pseudoharengus*) and blueback herring (*Alosa aestivalis*)—collectively known as “river herring”—are migratory sea‐run (i.e., anadromous) fishes that reproduce in lakes and rivers along the east coast of North America, but typically migrate to the Atlantic Ocean as juveniles to grow and reach sexual maturity before returning to their natal freshwater spawning grounds to reproduce (Loesch, 1987). River herring once supported an important commercial fishery, but spawning adult abundances have declined by 93% since 1970, and many spawning populations (hereafter “populations”) are now at historically low levels and are of increasing conservation concern (Hightower et al., 1996; Limburg & Waldman, 2009; Atlantic States Marine Fisheries Commission [ASMFC] 2012). Both species have broad distributions along the Atlantic coast (alewife: Labrador, Canada to North Carolina, USA; blueback herring: Gulf of St. Lawrence, Canada to Florida, USA), and resolving the range‐wide spatial scale of population genetic structure is an important component of conservation efforts and fishery management plans.

Recent genetic studies of alewife and blueback herring used polymorphic microsatellite markers to resolve the spatial scale of population genetic structure (McBride, Willis, Bradford, & Bentzen, 2014; Palkovacs et al., 2014), examine range‐wide patterns of hybridization (Hasselman et al., 2014), assess the influence of stocking activities on genetic structure (McBride, Hasselman, Willis, Palkovacs, & Bentzen, 2015), and determine the origin of river herring bycatch in commercial fisheries (Hasselman et al., 2016). Palkovacs et al. (2014) used data from microsatellites to reveal that US alewife populations (*n* = 21) were nested within three regional genetic stocks (Northern New England, Southern New England, and Mid‐Atlantic), whereas US blueback herring populations (*n* = 21) were nested within four regional genetic stocks (Northern New England, Southern New England, Mid‐Atlantic, and South Atlantic), with similar but not identical boundaries. Hasselman et al. (2016) also found that these same data had sufficient statistical power to confidently assign river herring bycatch in commercial fisheries to regional genetic stocks. Given their propensity for natal philopatry, the conservation and management of river herring requires a “population‐level” approach, and there is a need for molecular tools that can resolve population genetic structure at spatial scales finer than regional genetic stock. Moreover, for anadromous fishes, such as river herring, that migrate substantial distances across jurisdictional boundaries and are subject to capture as bycatch in mixed‐stock fisheries, a method that generates portable genetic data that can be easily shared and allows unambiguous assignment of individuals to population of origin is an important conservation and management tool (Clemento, Crandall, Garza, & Anderson, 2014; Morin et al., 2004; Starks, Clemento, & Garza, 2016).

Single nucleotide polymorphisms (SNPs) are bi‐allelic markers, ubiquitous in the genome of most species (Morin et al., 2004), that are relatively simple to genotype and provide data that are easily portable between laboratories and instruments. Recent higher‐throughput SNP genotyping technologies allow samples to be processed efficiently and in a cost‐effective manner (Clemento, Abadía‐Cardoso, Starks, & Garza, 2011; Larson, Seeb, Pascal, Templin, & Seeb, 2014; Seeb, Pascal, Ramakrishnan, & Seeb, 2009). SNP marker data have utility for a variety of ecological and evolutionary questions, and a suitable number of SNPs have been demonstrated to provide sufficient statistical power for resolving the spatial scale of population genetic structure in anadromous fishes (Clemento et al., 2014; Narum et al., 2008; Starks et al., 2016) and identifying pedigree relationships (Anderson & Garza, 2006). SNP data are also useful for assignment of individuals of unknown provenance to population of origin, often called genetic stock identification (GSI), and can be particularly informative when some of those SNPs have been affected by divergent selection between populations (Ackerman et al., 2011; Nielsen et al., 2012).

Here, we describe the development of two sets of 96 SNP assays, one specific to alewife and the other to blueback herring. These SNP panels are suitable for resolving range‐wide population genetic structure and have applications for GSI, investigating patterns of hybridization and introgression, and addressing issues of ecological and evolutionary relevance in a conservation and fisheries management framework. We used samples collected from across the ranges of both species for SNP discovery to minimize ascertainment bias (Albrechtsen, Nielsen, & Nielsen, 2010; Clark, Hubisz, Bustamante, Williamson, & Nielsen, 2005) and assess the power of the SNP data to accurately resolve previously described genetic stock structure for both species. The SNPs described herein will provide more power for population genetic investigations, enable higher throughput genotyping than with microsatellites, and allow for more effective data sharing across laboratories and management agencies.

## 2 MATERIALS AND METHODS

### 2.1 Sample collection

**Figure 1:**
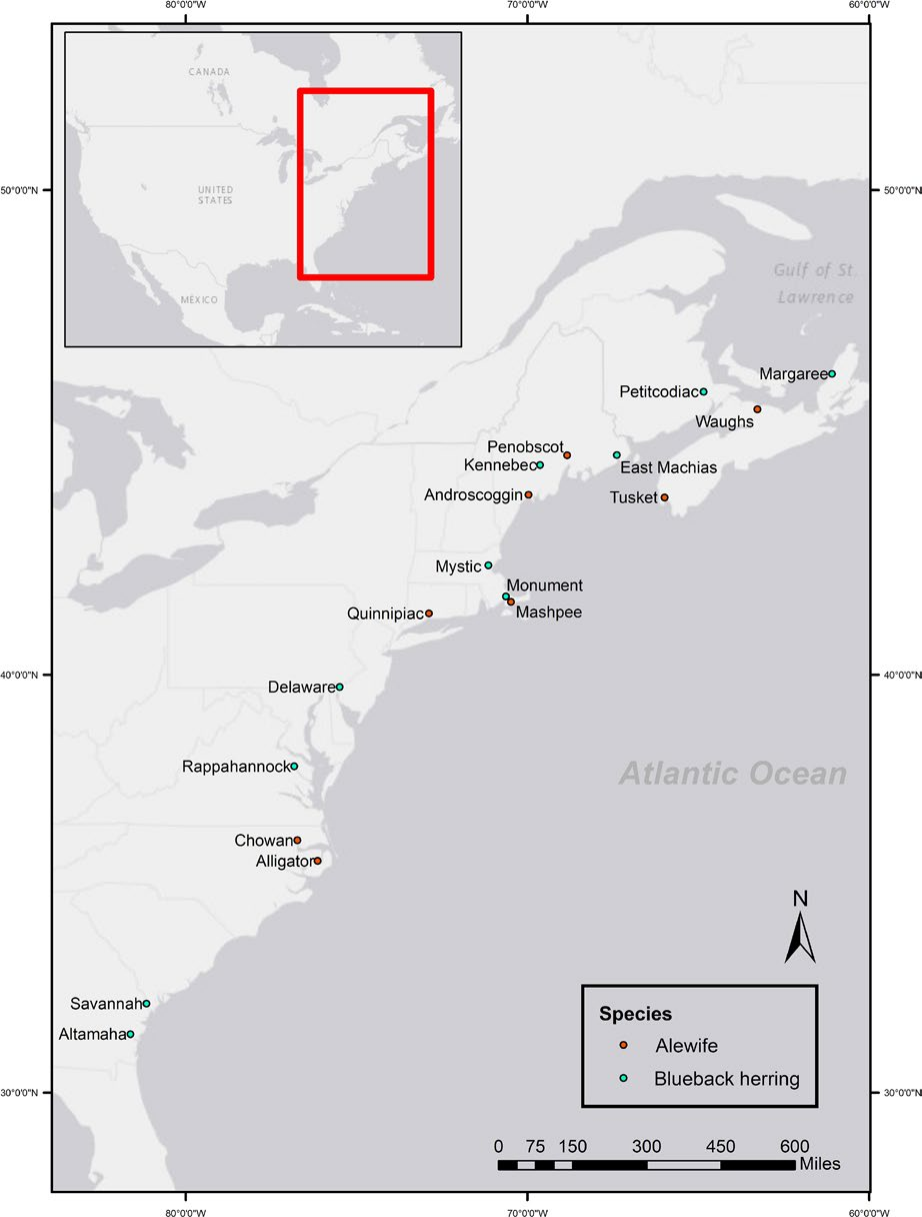
Map of sampling locations for alewife and blueback herring. Sampling locations are indicated by diamonds with associated river names

Muscle plugs or fin tissue was obtained from alewife and blueback herring captured across the species’ ranges (Table 1, Figure 1). All samples were obtained from adult fish and were collected in freshwater. Tissue was preserved in 95% ethanol until DNA extraction.

**Table 1:** Summary statistics of SNP assays in validation populations of (a) alewife and (b) blueback herring. Sample size consists of samples included in analyses. *H*_E_ is unbiased, expected heterozygosity, *H*_O_ is observed heterozygosity, No. of alleles is the mean number of alleles per locus in that population. Mean minor allele freq. is the frequency of the minor allele in the Quinnipiac River for alewife and the Monument River for blueback herring

Population	Sample size	Loci typed	*H*_E_	*H*_O_	No. of alleles	Percent polymorphic loci	Mean minor allele freq.
(a)							
Waughs	27	92	0.259	0.259	1.92	92.4	0.199
Tusket	45	92	0.265	0.265	1.95	94.6	0.206
Penobscot	44	93	0.267	0.264	1.96	95.7	0.213
Androscoggin	47	93	0.240	0.233	1.92	92.5	0.201
Mashpee	40	93	0.262	0.265	1.95	94.6	0.202
Quinnipiac	36	93	0.265	0.257	1.94	93.5	0.190
Chowan	41	93	0.216	0.223	1.88	88.2	0.180
Alligator	43	92	0.225	0.223	1.83	82.6	0.186
(b)							
Margaree	40	93	0.294	0.275	1.97	97.8	0.230
Petitcodiac	27	93	0.266	0.296	1.86	86.0	0.211
East Machias	45	94	0.298	0.295	1.98	97.9	0.238
Kennebec	47	96	0.301	0.305	1.99	97.9	0.240
Mystic	44	96	0.285	0.290	1.96	95.8	0.215
Monument	47	96	0.282	0.288	1.91	90.6	0.210
Delaware	47	95	0.290	0.290	1.97	96.8	0.224
Rappahannock	42	96	0.302	0.313	1.98	97.9	0.234
Savannah	47	96	0.275	0.267	1.95	94.8	0.251
Altamaha	47	96	0.277	0.281	1.95	94.8	0.247

### 2.2 SNP discovery and assay development

Tissue samples were removed from ethanol and air‐dried before extracting genomic DNA using DNeasy 96 Blood and Tissue kits with a BioRobot 3000 (Qiagen, Inc.). To identify potential SNPs for alewife and blueback herring, we used double digest Restriction Associated DNA sequencing (ddRADseq), a genome‐reduction technique that uses two restriction enzymes to create DNA fragments with identical fixed endpoints for annealing sequencing adapters (Peterson, Weber, Kay, Fisher, & Hoekstra, 2012). To ensure range‐wide coverage and reduce the risk of ascertainment bias, samples chosen for SNP discovery included individuals from at least one population from each of the regional genetic stocks for alewife and blueback herring previously identified by Palkovacs et al. (2014).

We performed ddRADseq library construction, sequencing, and SNP identification separately for each species, following the same protocol. Undiluted genomic DNA from 48 alewife from 18 populations and 12 blueback herring from four populations (Table S1), representative of genetic lineages throughout the species’ geographic ranges (McBride et al., 2014; Palkovacs et al., 2014) was digested using two restriction enzymes—Sph1 and EcoR1. Next, we performed size selection for 350‐bp fragments using the Pippin Prep system (Sage Science, Inc.). Following the addition of adapters, sequencing was performed on a MiSeq instrument (Illumina Inc.). We used two 600‐cycle sequencing reactions with paired‐end reads for alewife and a single such sequencing reaction for blueback herring. Sequence data from each species were processed, with homologous reads identified and SNPs called, using Stacks (Catchen, Hohenlohe, Bassham, Amores, & Cresko, 2013). Loci were selected for assay design by identifying sequences with a single SNP that also met the following three criteria: (1) all three genotypes (both homozygotes and the heterozygote) were observed, (2) a minimum of 20 sequence reads per allele for alewife and 15 reads for blueback herring were detected, and (3) the sequence did not share high similarity (>80%) with any other sequence selected from Stacks when global alignment was evaluated using BLAST (Altschul, Gish, Miller, Myers, & Lipman, 1990). These criteria were used to choose unique and sufficiently polymorphic target loci for the development of SNPtype genotyping assays (Fluidigm Corporation).

A total of 166 SNPs in alewife and 141 SNPs in blueback herring were chosen for SNPtype assay (Fluidigm) design. Assays were evaluated for consistency and polymorphism by genotyping 382 alewife samples from eight populations and 474 blueback herring from 10 populations throughout the species ranges (Figure 1; Table 1). SNP genotyping was performed with 96.96 Dynamic Genotyping Arrays on an EP1 Genotyping system (Fluidigm), which combines 96 DNA samples with 96 assays for a total of 9,216 reactions on each nanofluidic array. SNP genotypes were called with the Fluidigm SNP Genotyping software package.

Assays were first evaluated for their ability to produce clearly and consistently distinct clusters of genotypes. Loci were excluded that produced ambiguous genotypes or for which all validation samples appeared to have either homozygote or heterozygote genotypes, indicating null alleles or a lack of Mendelian inheritance. Sets of 96 well‐performing assays were then retained for the final alewife and blueback herring SNP panels. Details of these SNP genotyping assays, including target polymorphism, primer/probe sequences, and database accession numbers are in Table S2 (alewife) and Table S3 (blueback herring). Additionally, as there were more than 96 high‐quality blueback herring loci remaining at this stage, minor allele frequencies in the validation populations were estimated and used as a proxy for the expected power of the markers in pedigree reconstruction applications (Anderson & Garza, 2006) and used to choose the final panel of 96 assays.

Following genotyping, samples with missing data for 10 or more loci were excluded from further analyses (Table 1). Of the 382 alewife samples genotyped, 59 were excluded from validation analyses due to missing data. For the 474 blueback herring samples genotyped, the number excluded was 41. Allele frequencies, expected (*H*_E_) and observed heterozygosity (*H*_O_) for each locus were estimated using the Microsatellite Toolkit (v.3.1; Park, 2001). Concordance with expectations of Hardy–Weinberg and linkage (gametic phase) equilibria was determined with GenePop (v.4.2; Rousset, 2008) using an alpha value *p *= .05.

To evaluate the utility and performance of the two sets of SNP assays for GSI, self‐assignment analyses were conducted with GeneClass 2.0 (Piry et al., 2004), and the proportion of accurately assigned individuals per population and regional reporting group estimated. To evaluate utility for resolving range‐wide population structure, *F*_ST_ values were estimated for all pairs of populations within each species using Genetix (v.4.05; Belhkir, Borsa, Chikhi, Raufaste, & Bonhomme, 1996–2004). Model‐based clustering was performed with *structure* (Pritchard, Stephens, & Donnelly, 2000) without prior location information and using the admixture and correlated allele frequencies model. Clustering was evaluated for hypothesized numbers of genetic groups, *K *= 2–6, with 10 iterations at each value of *K*. Plots were generated using CLUMPP (Jakobsson and Rosenberg, 2007) and DISTRUCT (Rosenberg, 2004). Additionally, discriminate analysis of principal components (DAPC) (Jombart, 2008; Jombart, Devillard, & Balloux, 2010), implemented in the package *adegenet* (version 2.0.1) in R 3.3.1 (R Core Development Team 2016), was used to identify population structure.

## 3 RESULTS

Sequencing yielded approximately 54 million reads in alewife and 33 million reads in blueback herring. After filtering with Stacks software (Catchen et al., 2013), 33,868 and 19,809 unique loci were found for alewife and blueback herring, respectively. Of these, 4,934 loci containing a single SNP were identified in alewife and 2,810 loci in blueback herring. From the loci selected for SNPtype assay design, two unique sets (one for each species) containing 96 SNPs each were used for validation (Tables S2 and S3). Three loci (Aps_2060, Aps_4755 and Aps_8787) did not show consistent clustering in the alewife validation populations and were removed from further analyses.

Genetic variation of the validated SNPs was generally lower in alewife (Table S4) than in blueback herring (Table S5), with mean observed heterozygosity (*H*_O_) of 0.290 in blueback herring versus *H*_O_ of 0.249 in alewife. Similarly, the mean minor allele frequency across blueback herring populations was 0.230, whereas it was 0.197 in alewife. While still adequate for parentage analyses (Anderson & Garza, 2006), diversity values are generally lower than in other anadromous species, such as Chinook salmon (e.g., Clemento et al., 2011).

There were 15 loci that deviated from HWE expectations in one alewife population, one locus that deviated in two populations (Aps_14730), and one locus in four populations (Aps_5844) (Table S4). For blueback herring, 21 loci were not in HWE in one population, four loci in two populations (Aae_2120, Aae_4985, Aae_5780, Aae_7796), one locus in three populations (Aae_8427), and one locus in four populations (Aae_5563). Significant linkage disequilibrium was identified in 58 (of 4,278) pairs of loci in alewife and 88 (of 4,562) pairs of loci in blueback herring. These are less than the numbers expected by chance alone, and no geographic pattern in the significant values was apparent for either species.

The majority of both blueback herring and alewife individuals were accurately assigned to their population and reporting group of origin in the self‐assignment analyses. For alewife, 67% of all fish were correctly assigned to population of origin and 93% to reporting group of origin (Table 2a) when no probability criterion was used, and 83% to population and 97% to reporting group, when a 90% probability criterion was applied (Table 2b). For blueback herring, assignment accuracy was similar, with 67% accurately assigned to population of origin and 96% assigned to reporting group of origin with no probability criterion (Table 2c) and 79% to population and 98% to reporting group, when a 90% probability criterion was applied (Table 2d). Self‐assignment for alewife was less accurate in northern populations than in the south, corresponding to increased geographic distance between rivers in the south, whereas there was no discernible pattern with blueback herring.

**Table 2:** Accuracy of leave‐one‐out self‐assignment analyses to population and regional stock for alewife (a) without applying a probability criterion (i.e., all individuals assigned) and (b) with a 90% criterion. Blueback herring are also assigned (c) without applying a probability criterion (d) with a 90% criterion

True population	Waughs	Tusket	Androscoggin	Penobscot	Mashpee	Quinnipiac	Chowan	Alligator	Prop. assigned population	Prop. assigned‐regional stock
(a) Alewife, no probability criterion										
Waughs	**18**	4			2	2		1	.667	.815
Tusket	3	**38**	1	1	1	1			.844	.911
Androscoggin			**34**	13					.723	1.000
Penobscot		1	16	**26**	1				.591	.955
Mashpee	4			1	**25**	9	1		.625	.850
Quinnipiac	2				6	**27**		1	.750	.917
Chowan						1	**27**	13	.659	.976
Alligator							22	**21**	.488	1.000
(b) Alewife, 90% probability criterion										
Waughs	**12**	2			1				.800	.933
Tusket	1	**32**							.970	1.000
Androscoggin			**11**	3					.786	1.000
Penobscot			4	**14**	1				.737	.947
Mashpee	2				**18**	2			.818	.909
Quinnipiac					4	**19**			.826	1.000
Chowan							**13**	4	.765	1.000
Alligator							2	**10**	.833	1.000

True population	Margaree	Petitcodiac	East Machias	Kennebec	Mystic	Monument	Delaware	Rappahannock	Savannah	Altamaha	Prop. assigned population	Prop. assigned to regional stock
(c) Blueback, no probability criterion												
Margaree	**23**	1	7	6	1			2			.575	.925
Petitcodiac		**27**									1.000	1.000
East Machias	3		**25**	15		1		1			.556	.956
Kennebec	4		9	**31**	1			2			.660	.936
Mystic					**38**	5	1				.864	.977
Monument					5	**42**					.894	1.000
Delaware				1			**23**	20	1	2	.489	.915
Rappahannock	1			3	1		23	**14**			.333	.881
Savannah									**33**	14	.702	1.000
Altamaha									14	**33**	.702	1.000
(d) Blueback, 90% probability criterion												
Margaree	**11**		1		1						.846	.923
Petitcodiac		**23**									1.000	1.000
East Machias			**5**	5		1					.455	.909
Kennebec			2	**14**				1			.824	.941
Mystic					**30**	3					.909	1.000
Monument					1	**32**					.970	1.000
Delaware							**6**	8			.429	1.000
Rappahannock				1			8	**3**			.250	.91
Savannah									**17**	6	.739	1.000
Altamaha									3	**13**	.813	1.000

Bold = assignment to correct population of origin.

Comparisons across alewife populations found generally low differentiation across the species range, with significant *F*_ST_ values ranging from 0.006 to 0.140. The highest *F*_ST_ values were between the southernmost populations (Alligator and Chowan) and populations in the Northern New England reporting group (Penobscot and Androscoggin), with somewhat less differentiation seen between these populations and the northernmost ones in Canada (Waughs and Tusket) (Table 3a). Pairwise *F*_ST_ was nonsignificant only between the Alligator and Chowan Rivers. Pairwise *F*_ST_ values in blueback herring showed a similar pattern of modest genetic differentiation across the range, with significant values ranging from 0.004 to 0.150. The two southernmost populations were again the most genetically distinct when compared to all other validation populations, with almost all values of *F*_ST_ > 0.1, whereas none of the other values exceeded 0.1 (Table 3). Pairwise *F*_ST_ was significantly different from zero between all pairs of populations, except those from the Delaware and Rappahannock Rivers.

**Table 3:** Pairwise *F*_ST_ values. Significance assessed with 200 permutations

Population	Tusket	Androscoggin	Penobscot	Mashpee	Quinnipiac	Chowan	Alligator
Alewife							
Waughs	0.029	0.075	0.060	0.030	0.034	0.083	0.075
Tusket		0.067	0.055	0.061	0.059	0.088	0.080
Androscoggin			0.006	0.064	0.067	0.140	0.124
Penobscot				0.050	0.051	0.131	0.115
Mashpee					0.015	0.077	0.066
Quinnipiac						0.075	0.064
Chowan							***0***.***000***

Population	Petitcodiac	East Machias	Kennebec	Mystic	Monument	Delaware	Rappahannock	Savannah	Altamaha
Blueback herring									
Margaree	0.049	0.010	0.017	0.063	0.056	0.039	0.035	0.122	0.109
Petitcodiac		0.048	0.054	0.092	0.075	0.091	0.086	0.150	0.138
East Machias			0.004	0.069	0.063	0.046	0.043	0.134	0.119
Kennebec				0.065	0.062	0.042	0.040	0.131	0.113
Mystic					0.025	0.057	0.057	0.123	0.112
Monument						0.078	0.075	0.123	0.116
Delawar e							***−0***.***002***	0.087	0.075
Rappahannock								0.087	0.076
Savannah									0.006

Bold italics ***= ***nonsignificant value.

**Figure 2:**
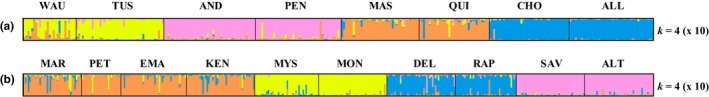
Bayesian clustering analyses for (a) alewife and (b) blueback herring. The vertical lines represent fractional ancestry of individual fish partitioned into *K *= 4 clusters, as indicated by colors. Alewife: WAU, Waughs River; TUS, Tusket River; AND, Androscoggin River; PEN, Penobscot River; MAS, Mashpee River; QUI, Quinnipiac River; CHO, Chowan River; ALL, Alligator River. Blueback herring: MAR, Margaree River; PET, Petitcodiac River; EMA, East Machias River; KEN, Kennebec River; MYS, Mystic River; MON, Monument River; DEL, Delaware River; RAP, Rappahannock River; SAV, Savannah River; ALT, Altamaha River

**Figure 3:**
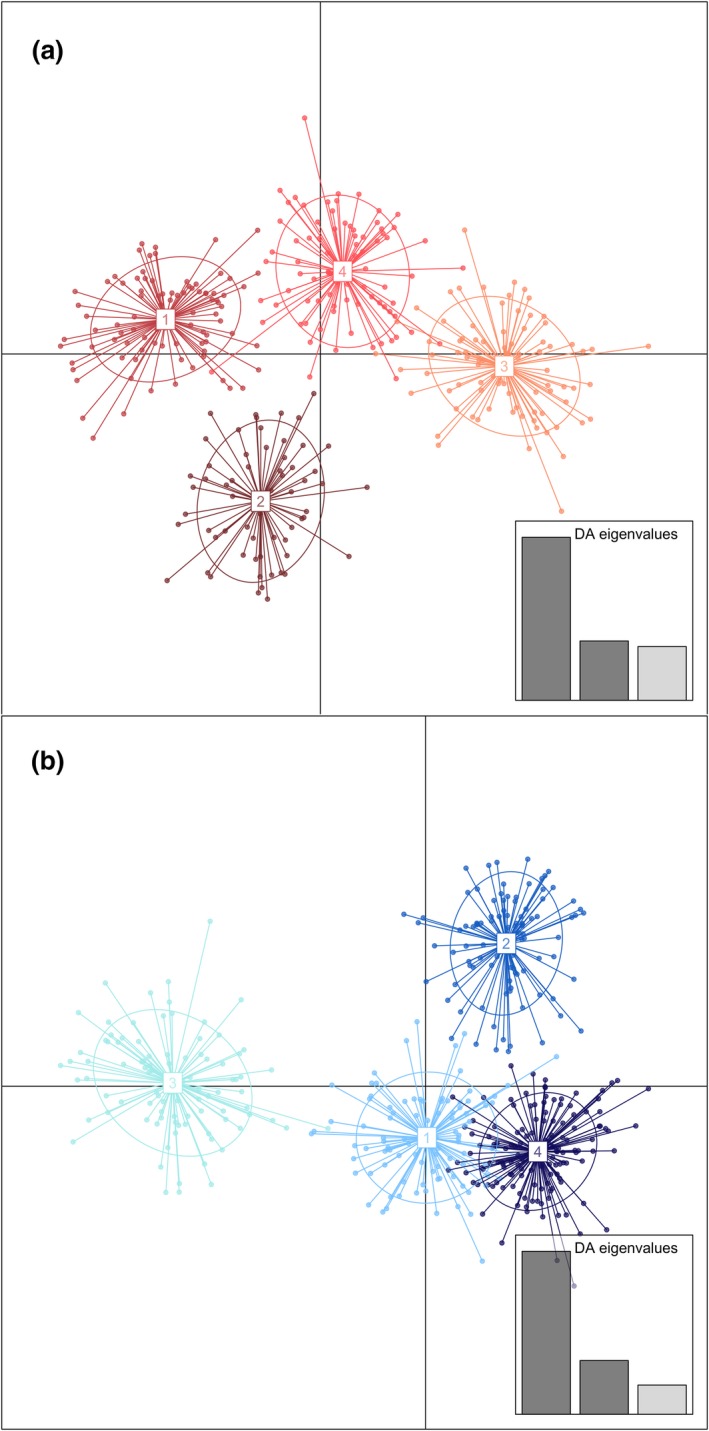
Scatter plots of Discriminant Analysis of Principal Components (DAPC, Jombart, 2008) for (a) alewife and (b) blueback herring. In both species, the lowest BIC values corresponded with clusters of *K *= 4, and partitions populations geographically. The eigenvalues for the first three principal components are indicated in the insets for each species. The clusters for alewife represent (1) AND and PEN, (2) WAU and TUS, (3) CHO and ALL, and (4) MASH and QUI. For blueback herring, the clusters represent (1) DEL and RAP, (2) MYS and MON, (3) SAV and ALT, and (4) MAR, PET, EMA, and KEN. Population codes are as in Fig. 2

Model‐based clustering analysis with *structure* for the eight populations of alewife found that, at *K *= 2, the two southernmost populations, Chowan and Alligator Rivers, cluster together (Fig. S1), consistent with their higher *F*_ST_ values with northern populations and the nonsignificance between them. At *K *= 3, the Penobscot and Androscoggin River populations cluster together. At *K* = 4, the northernmost Waughs and Tusket populations formed a distinct cluster (Figure 2). DAPC identified the same four clusters (Figure 3a). Clustering analyses with the 10 blueback herring populations at *K* = 2 again found that the two southernmost populations, the Savannah and Altamaha Rivers, formed a distinct cluster (Fig. S1). At *K* = 3, two proximate populations in the middle of the range, the Mystic and Monument Rivers, separated as a distinct cluster. At *K* = 4, the four northernmost populations separated (Figure 2), while at higher values of *K*, most individuals in multiple populations began to separate fractionally into different clusters, indicating that the analysis had exceeded the most likely value of *K*. Clustering with DAPC again identified four distinct groups (Figure 3b), which mostly corresponded to the clusters found by *structure*.

## 4 DISCUSSION

Molecular genetic data and analysis have become a critical component of biological investigation and conservation for migratory species, particularly anadromous fishes that are harvested and often subject to multijurisdictional management (Clemento et al., 2014; Hasselman et al., 2016; Palkovacs et al., 2014). We describe here validated SNP assays for alewife and blueback herring that provide power for multiple applications, including GSI across the species’ ranges, as well as pedigree reconstruction and phylogeography.

Self‐assignment analyses with both alewife and blueback herring populations demonstrated clear delineation between regional genetic stocks previously identified using microsatellite data (Palkovacs et al., 2014). Alewife validation populations displayed differentiation with these SNP assays that mirror the regional population genetic structure previously identified by Palkovacs et al. (2014) and expand the utility of genetic identification into the northern portion of the species range. Pairwise *F*_ST_ values revealed significant differentiation between all sets of populations except the Chowan and Alligator Rivers (Table 3), which are geographically proximate and tributaries of the same coastal estuary (Albemarle Sound). Two‐thirds of alewife samples assigned to their correct population/river basin of origin, but the proportion of accurate assignments by population ranged between 49% and 84% (Table 2) when no probability criterion was applied. When such a criterion was applied, the overall proportion of accurate assignments increased substantially (82%), and the proportion per population ranged from 74% to 97%, but nearly half of the samples remained unassigned, emphasizing the lack of fine‐scale differentiation and population structure in alewife. When self‐assignment was evaluated at the scale of the previously reported regional genetic stocks, accuracy was much higher, with 93% of samples assigned accurately to regional stock of origin and the accuracy per population of assignment to reporting unit ranged from 82% to 100% without a probability criterion and 91% to 100% with a probability criterion.

Model‐based clustering results from *structure* were consistent with the self‐assignment and DAPC results for alewife and found that populations within the same regional genetic stock generally clustered together (Figure 2a and 3a, Table 2a,b). The two southernmost populations separated first, followed by the two Southern New England populations (Quinnipiac and Mashpee Rivers; Palkovacs et al., 2014), at *K* = 3. The two Northern New England populations and the two northernmost populations, in Canada, separated at higher *K* in some iterations. The two northernmost populations are both in Nova Scotia but geographically distinct, as one (Waughs) is in the Gulf of the Saint Lawrence Seaway and the other (Tusket) borders the Bay of Fundy, where strong population structure has been identified in other anadromous species: America shad (*A. sapidissima,* Hasselman et al., 2010, 2013), Atlantic salmon (*Salmo salar,* McConnell et al., 1997; Verspoor et al., 2002; Spidle et al., 2003), striped bass (*Morone saxatilis,* Bradford et al., 2012), and rainbow smelt (*Osmerus mordax*, Coulson et al., 2014). Previous work on alewife (McBride et al., 2014) found elevated levels of differentiation within the Bay of Fundy, as well as different life history traits and homing patterns between inner Bay of Fundy rivers and those outside the Gulf of Maine in North America.

For blueback herring, pairwise *F*_ST_ values revealed significant differentiation between all sets of populations except the Delaware and Rappahannock Rivers (Table 3). This is consistent with movement between the Delaware River and Chesapeake Bay (likely via the Chesapeake and Delaware Canal), as has been shown by otolith microchemistry (Turner, Limburg, & Palkovacs, 2015). The proportion of blueback herring correctly self‐assigned to population of origin was similarly variable, ranging from 33% to 100% without a probability criterion (Table 2). Using a probability criterion increased overall accuracy of assignments (72%), with a range of 43%–100% per population. Geographic proximity and population connectivity appear to explain many of the misassignments. For example, a high frequency of misassignments involved the Delaware and Rappahannock Rivers, which are connected via the Chesapeake and Delaware Canal, and the Savannah and Altamaha, which are geographically proximate (Figure 1). Similar to alewife, assignment to previously reported regional stocks was much more accurate, with overall assignment of 96% without a probability criterion and 97% with a probability criterion (Table 2).

The *structure* clustering results with blueback herring populations again mirrored the self‐assignment and DAPC results (Figure 2b and 3b, Table 2c,d). Although the validation samples encompass most of the species range, the spatial distribution of populations is uneven, and the proximate populations consistently grouped. At *K* = 2, the southernmost populations, in the Altamaha and Savannah Rivers, clustered separately (Fig S1), whereas at *K* = 3, the proximate Southern New England populations separated. At *K* = 4, the four regional genetic stocks within the US range, previously identified by Palkovacs et al. (2014), were recovered (Figure 2b); however, the northernmost populations (Margaree and Petitcodiac) grouped with the Northern New England populations (Kennebec and East Machias). At *K* = 5, the Petitcodiac separated, whereas the Margaree continued to group with Northern New England (Fig. S1).

The 96 locus SNP set for blueback herring provided clustering concordant with the genetic stocks previously identified with microsatellite markers, and the four northernmost populations, which had not previously been evaluated together, formed a distinct cluster. The alewife SNP panel extends the geographic range for GSI, and also recovers clusters consistent with the regional genetic stocks found with microsatellites, yet the ability for the assays to discriminate population structure at small spatial scales, especially among rivers exchanging frequent migrants, may prove difficult.

Data from SNP assays are unambiguous and easily portable between laboratories. Generating these markers using high‐throughput sequencing of genomic DNA enhances our ability to confidently distinguish populations of alewife and blueback herring that are genetically distinct across both species’ ranges. This ability to identify stock of origin for fish caught at sea is critical for management of populations experiencing diminished spawning returns and of increasing conservation concern (Hasselman et al., 2016). Using these new tools, samples from mixed‐stock assemblages can be quickly and efficiently genotyped, allowing new insights into marine movement patterns and impacts of marine fisheries.

## AUTHOR CONTRIBUTIONS

D. S. Baetscher conducted laboratory work and performed analyses. D. J. Hasselman collected samples and performed analyses. K. Reid performed analyses. E. P. Palkovacs collected samples and obtained funding. J. C. Garza performed analyses and obtained funding. All authors participated in study design and manuscript composition.

## CONFLICT OF INTEREST

None declared.

## DATA ACCESSIBILITY

Genotype data for all fish with all assays, FASTA files with consensus sequences from all ddRAD loci with at least one SNP, as well as haplotype calls from all combinations of locus and individual are in the DRYAD digital repository (doi:10.5061/dryad.v4q83).
