# Supplementary material for: Discovery and characterization of single nucleotide polymorphisms in two anadromous alosine fishes of conservation concern
Source: Ecol Evol. 2017 Jul 18;7(17):6638–48. doi: 10.1002/ece3.3215 (PMC5587496; doi:10.1002/ece3.3215)

Figure S1: Bayesian clustering analyses for a) alewife and b) blueback herring. Lines represent fractional ancestry of individual fish partitioned into *K* = 2 to 6, clusters as indicated by colors. Alewife: WAU-Waughs River, TUS-Tusket River, AND-Androscoggin River, PEN-Penobscot River, MAS- Mashpee River, QUI-Quinnipiac River, CHO-Chowan River, ALL-Alligator River. Blueback herring: MAR-Margaree River, PET-Petitcodiac River, EMA-East Machias River, KEN-Kennebec River, MYS-Mystic River, MON-Monument River, DEL-Delaware River, RAP- Rappahannock River, SAV-Savannah River, ALT-Altamaha River.

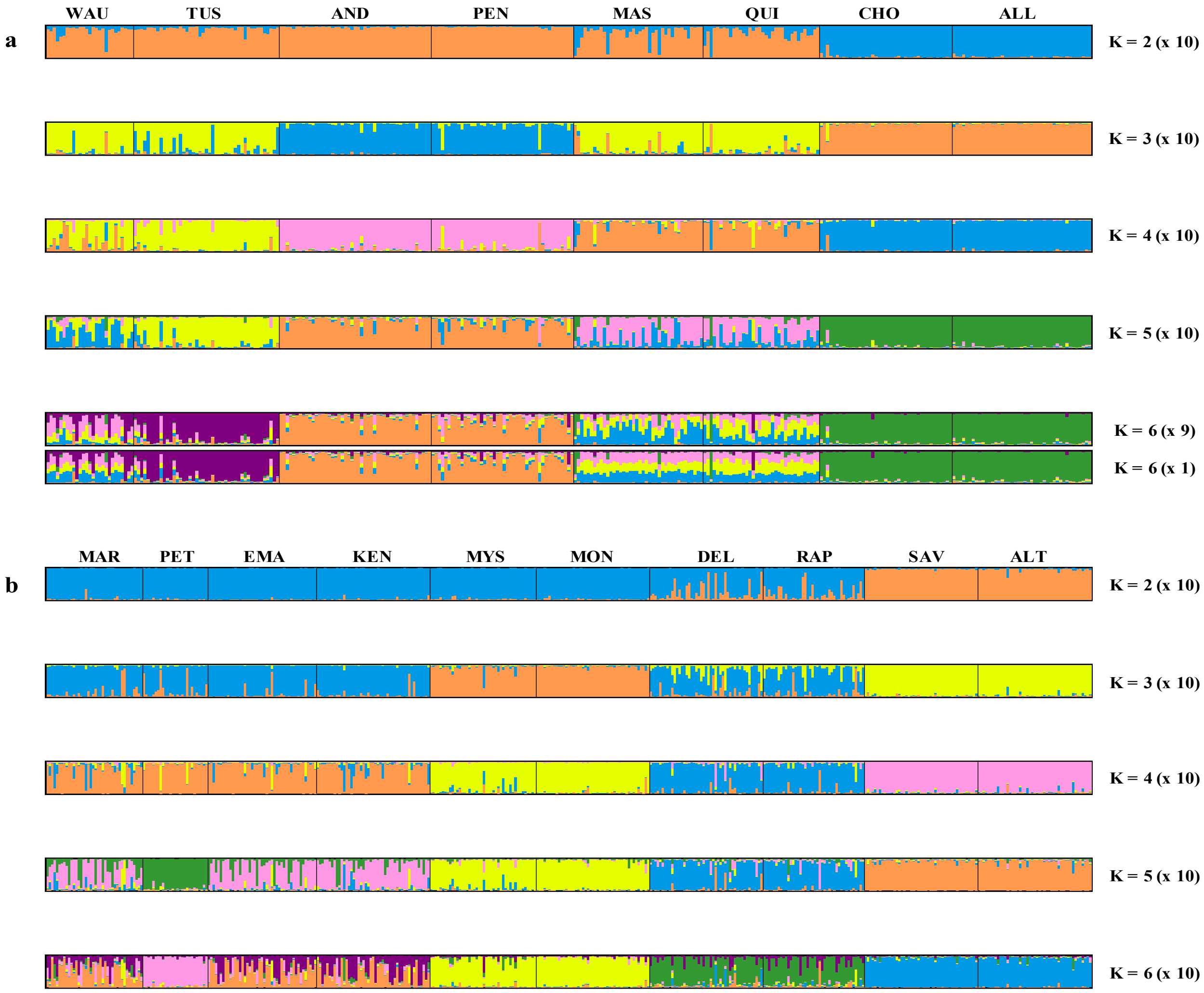

Supplement: Supplementary file 1 [file ECE3-7-6638-s001.pdf]
