# Supplementary material for: Discovery and characterization of single nucleotide polymorphisms in two anadromous alosine fishes of conservation concern
Source: Ecol Evol. 2017 Jul 18;7(17):6638–48. doi: 10.1002/ece3.3215 (PMC5587496; doi:10.1002/ece3.3215)
Supplement: Supplementary file 2 [file ECE3-7-6638-s002.pdf]

Table S1. Populations of alewife and blueback herring used in SNP discovery with double-digest RAD sequencing, with number of samples for each population. For non-anadromous populations, the basin in which it is located is also noted.

Population	Connection to Atlantic Ocean	No. of samples
**Alewife**		
Miramichi River		2
Medway River		2
Argyle Brook		2
Shubenacadie River		2
Saint John River		2
Damariscotta River		2
Sewell Pond		2
St. George River		2
Hudson River		2
Rappahannock River		2
Lake Ontario		2
Ostico Lake	Landlocked - NY Finger Lakes	2
Quonnipaug Lake	West River, CT - Long Island Sound	4
Rogers Lake	Lieutenant River, CT - Connecticut River	4
Pattagansett Lake	Pattagansett River, CT - Long Island Sound	4
Bride Brook	Long Island Sound	4
Dodge Pond	Pattagansett River, CT - Long Island Sound	4
Mill Brook	Connecticut River	4
**Blueback**		
East Machias River		3
Gilbert-Stuart River		3
Connecticut River		3
Savannah River		3
