# Supplementary material for: Discovery and characterization of single nucleotide polymorphisms in two anadromous alosine fishes of conservation concern
Source: Ecol Evol. 2017 Jul 18;7(17):6638–48. doi: 10.1002/ece3.3215 (PMC5587496; doi:10.1002/ece3.3215)
Supplement: Supplementary file 3 [file ECE3-7-6638-s003.pdf]

Table S2. Details of all SNP genotying assays for alewife.

Assay name	Assay target	Primers (5'-3')	Probes (5'-3')	GenBank No.
Aps_1000	T/C	F: TCCCGTAAGGGTATAAGGCAAAA	FAM: GCATCATCTGTCCCCATAGGAT	
		R: TCTGTGTATGTACTCTTGGTTCATTCTTGATTAAA	VIC: GCATCATCTGTCCCCATAGGAC	
Aps_10095	A/G	F: GATTGCCGAAACAGGACATCT	FAM: CCTTCATGGATCAGTGTTCTTGGA	
		R: CAAGCTGGCGACTTAGCCC	VIC: CTTCATGGATCAGTGTTCTTGGG	
Aps_10259	C/A	F: AGGCCGTATCACTTTCTATCGT	FAM: TGTGATCAAATACACAATATTCAAACCAGAAC	
		R: ACTGACTTATCTAGTTAAATAGTGTCAAAGTATAGTATAG	VIC: TGTGATCAAATACACAATATTCAAACCAGAAA	
Aps_10291	T/A	F: AAAGCACAGGGAACTTAAAAAGAGT	FAM: GTTAACGGTTAAGTTGCCATCTGTT	
		R: TCCGACATGCTTTTATATAAATAGTAATGGATGACTG	VIC: AGTTAACGGTTAAGTTGCCATCTGTA	
Aps_10338	C/T	F: AGACACCATAGCCTGCCAA	FAM: CAGAACATATCTCAAACCGGACTTG	
		R: CCCCGTTGCATGGTCTCATC	VIC: AACAGAACATATCTCAAACCGGACTTA	
Aps_10554	G/T	F: TCATCATAGACAAATGGCAAACACA	FAM: ACAAACAATTTAGTCATTCTATGATTTAGCCG	
		R: CCAATAAGTAATTTCATAGTGGCAGCATTAACT	VIC: AACAAACAATTTAGTCATTCTATGATTTAGCCT	
Aps_11191	T/C	F: GCCTTTTACAGTAAACATGCAACA	FAM: AACAGTAACTCATGGAGCAGTGT	
		R: CTCTATAAAATTAGAAAACCTCCCATTCTAGCAGT	VIC: ACAGTAACTCATGGAGCAGTGC	
Aps_11218	A/G	F: TGGTGTCCTTATGGCGGT	FAM: TGGAAAGCGGTTCATCACACT	
		R: ACACTCACTCACTCCCTCCCT	VIC: GGAAAGCGGTTCATCACACC	
Aps_11832	T/G	F: GCTCTCCAGTGCAAAGCT	FAM: TGAAATGGCGTATCAAATGAGACTCA	
		R: CTCCACAGCAGAGGATGAAGGT	VIC: GAAATGGCGTATCAAATGAGACTCC	
Aps_12281	A/T	F: CGGTCATTTTACTGCCTAGGTC	FAM: CTACATTACTTAATCTCTCCGACTAACACA	
		R: GGGAAGCATCTTTGGCACAGT	VIC: CTACATTACTTAATCTCTCCGACTAACACT	
Aps_12422	G/A	F: TGTGTTATGTACGTCATTCATCTGC	FAM: CCTCTCTGCTAGCTTGAGACAAAG	
		R: GGGAATAAAAAAAGAGAACATTATCCTCAAGCA	VIC: CCTCTCTGCTAGCTTGAGACAAAA	
Aps_139	A/T	F: AAATGTGTGCTAAACAGGTGAACT	FAM: CAGGTGAACTGAAGAAGAGGCA	
		R: GTGCCCTTGTTCTTGGAAGCA	VIC: CAGGTGAACTGAAGAAGAGGCT	
Aps_1475	A/T	F: AGTCTACAATCAAACAACAGCAACA	FAM: TCAATGGATCCATATGGTTAAGTCCAA	
		R: TGTCCCCATGCCAGCTTGA	VIC: TCAATGGATCCATATGGTTAAGTCCAT	
Aps_1607	C/T	F: ATCACCTGGGATTCCCAATTTG	FAM: GCGCTGCCAATGTGGATC	
		R: AGGCCAGACTGCTGTCCA	VIC: GCGCTGCCAATGTGGATT	
Aps_1667	G/A	F: CTTATTACTGCAGATGTGCTTCAGA	FAM: ACATCTTTACAAAGTGGCATTTTCCG	
		R: CAGAAGGCAAGGCAAGGCA	VIC: AATACATCTTTACAAAGTGGCATTTTCCA	
Aps_1762	G/T	F: TTGTAACTAACGACCTCCACCA	FAM: CCACCACATATTTACAAAAGGCCTC	

		R: CAACACCATGGCTGCTTAGCT	VIC: TCCACCACATATTTACAAAAGGCCTA
Aps_1929	G/A	F: GCAGGCCTAATTTCCAGTTTTCC	FAM: CGTTTCCGCAGAGTGTAACAC
		R: AGCTGGCCTCATTCTCACACTT	VIC: CCGTTTCCGCAGAGTGTAACAT
Aps_1991	A/T	F: CGGGCGGGCTTAGGA	FAM: AGGCCTAAATGTAGGCTATTTCTTTATCA
		R: ACTGAAACATTTTATATTAGTGGTAACATAAAAACGATGA	VIC: GTAGGCCTAAATGTAGGCTATTTCTTTATCT
Aps_2060	T/A	F: GTGGATGATGCTACATGGCT	FAM: ATGGCTTCACATCCCAGACAT
		R: CCTGTCAGAGAAAACTTGCCCA	VIC: TGGCTTCACATCCCAGACAA
Aps_2158	G/T	F: CTCAATTCAGTGGAAAGCGATTTT	FAM: ACGTCATCCCTGAGAGCATTAATC
		R: GGATGCTAGCTCGCTACAGGA	VIC: CATACGTCATCCCTGAGAGCATTAATA
Aps_2556	A/G	F: CAGCTTTACAGATAATTAGGGAGGG	FAM: GCGTCTTCTAATGAACATCAGAGCA
		R: CGGTGGAAAGAGGGTTCTCCATTA	VIC: CGTCTTCTAATGAACATCAGAGCG
Aps_257	A/T	F: GTATGGTTCAGTTATCATGATGGCA	FAM: GCATGATATTCAAACGAGTTGTGGT
		R: CACTGAATACAACTGCTGCCTGC	VIC: GCATGATATTCAAACGAGTTGTGGA
Aps_2866	A/C	F: ATGTGAGGACTAACTCAGCTGT	FAM: GCTGTAAAATCACTTAACAAAGACACTTCA
		R: CGGTGAGGCTGGTTTCTCTCA	VIC: GCTGTAAAATCACTTAACAAAGACACTTCC
Aps_290	T/C	F: AGAGGAAAGGGAGATGACTCATT	FAM: GTAGTGTTGCTGCATTAATCACAGA
		R: TTCCCTGAGGCATCTGCTTCT	VIC: GTAGTGTTGCTGCATTAATCACAGG
Aps_2925	G/A	F: CCCTGCACTTTGCTTTTGTTTC	FAM: CTCCCGTTTGTAATTCACACATACG
		R: TGGCAGGTCAGGATGACAATCT	VIC: TCTCCCGTTTGTAATTCACACATACA
Aps_3089	C/A	F: GAAGAATGTACAGTACAAGGCCA	FAM: GTGCTGTCCTCCTCTCTGTATAAAG
		R: CTCTGTGATGAATATCCCATCTGCATATACA	VIC: GTGCTGTCCTCCTCTCTGTATAAAT
Aps_3269	T/C	F: GCTTCTCAAAGGGTTTGTGAAGT	FAM: CGCTACCAAATCTTCTTCTGTCTCT
		R: GTTCAAACGCGCGGTCTCC	VIC: CGCTACCAAATCTTCTTCTGTCTCC
Aps_3344	C/T	F: GCCGTGTAAGCTGTGTTTATTTT	FAM: TCAGGCGTCACATCCTTCC
		R: TCAAGCCACAGCAGTCCCT	VIC: GTCAGGCGTCACATCCTTCT
Aps_3356	G/A	F: ACCAATGGCAAAATATACAGTCAGT	FAM: AGTCAGTGCTATGGAGAAACCG
		R: ACTGCCTTTTCCAGACCTCTGA	VIC: CAGTCAGTGCTATGGAGAAACCA
Aps_3404	G/C	F: CACTCTGCACTAAAATGTGCAGT	FAM: AAAATGTGCAGTCTTCTCACACAAC
		R: GCATGTACAGCAAGCTCCCC	VIC: CTAAAATGTGCAGTCTTCTCACACAAG
Aps_3478	C/A	F: CCTTTGTTTTGTTTTAGCCATTGGG	FAM: CTTGCAAATTCACAAAGGAACCAG
		R: CAAAGTTCATGGATATAGTACTGGCTATATTGCTAT	VIC: GCTTGCAAATTCACAAAGGAACCAT
Aps_3561	A/G	F: CAAGATGGCTTCCGGTTTAATAAAG	FAM: AATAAGTATTGCATAACGGCAATCACAA
		R: ACAATATTACTTTGCTTTCCATTTTATGATACAGCA	VIC: AATAAGTATTGCATAACGGCAATCACAG
Aps_3879	C/T	F: GTAAGCATCCTGACACAACCA	FAM: CCAGGTCAAGACCCACCAC
		R: GGGTGGACACCATCGTTGTC	VIC: CCAGGTCAAGACCCACCAT

Aps_3936	G/T	F: CCAATAAAGCTGCCATTGAACAAC	FAM: CGTTGGTGTCGTGTGCATTAC
		R: CCTTGGTTAGAACAGCGCCT	VIC: CGTTGGTGTCGTGTGCATTAA
Aps_4254	G/T	F: GGGAGGTCAGATTCTGTAAAATACC	FAM: GAAGGGGCAGAAACAAAAGACAC
		R: TCAGCACATGCGCTCTTTGC	VIC: GAAGGGGCAGAAACAAAAGACAA
Aps_4334	A/G	F: CATCGGAAGATGGAGACGTTT	FAM: GTTGCTATGAGGGTCTCCATCTT
		R: TCATTCAGGCCAGCAGTGGA	VIC: GTTGCTATGAGGGTCTCCATCTC
Aps_4413	C/A	F: GTGTTTCCTACAGTTGCCTTCA	FAM: CATTCCACATGCATCTAGCCAC
		R: TCATGTCTTGACAGGACGCACA	VIC: TCATTCCACATGCATCTAGCCAA
Aps_4437	A/G	F: ATTGCAAGTGTAAGCCACACA	FAM: ATTACTGCATCTTCCCTTGTCCT
		R: GCGCAAGTACTGTCCTACACCA	VIC: ACTGCATCTTCCCTTGTCCC
Aps_4755	G/C	F: CATTGTATCATTACACCCTCTCTGC	FAM: CACATACACATGACTTGGTCAATACAATC
		R: TGTGTTGTATGACGTTCTTAATGTCCTTGA	VIC: CACATACACATGACTTGGTCAATACAATG
Aps_4891	A/G	F: CCAACTGGATCTCACTTTTCTCA	FAM: TTCCCACTTTCTCTCACTTCTTTGAT
		R: GCAAAGATAACATAGAAACATAAAAACGTGAAATGTC	VIC: TCCCACTTTCTCTCACTTCTTTGAC
Aps_5039	A/G	F: ACTGGGAGACGTCACAGG	FAM: CAGGAGGCTAATTTCTATGATGAAGGT
		R: AGGGACCATCCCACATGTGTT	VIC: AGGAGGCTAATTTCTATGATGAAGGC
Aps_5087	G/A	F: TGTGGGATTTTAATCCAAACTTCAAGA	FAM: AGAACATGCGGTAGTTAGAGCATG
		R: TGTCATCTACTTGGGCTGCAGA	VIC: AAAGAACATGCGGTAGTTAGAGCATA
Aps_5166	G/A	F: AATTGAATAAGAGAGTATGTGGTCACAAA	FAM: GAGAGTATGTGGTCACAAAAAGCC
		R: TGCCGTTTGAACATTACCTACAGAGT	VIC: AGAGAGTATGTGGTCACAAAAAGCT
Aps_5177	T/G	F: ATCACGGACCATTTCATTTCACA	FAM: AGGTCTGAAACACTTGCACTGTTA
		R: GCGGCGGCATTTCAGCA	VIC: GGTCTGAAACACTTGCACTGTTC
Aps_521	G/A	F: TGAGGTGAGGCTTTGCCTA	FAM: GCCTAAACATTCAACATTATTTGCTTTGC
		R: TCTTTATCTAGAGTATAATAGTCTACTTGAGTACAAGGAT	VIC: GCCTAAACATTCAACATTATTTGCTTTGT
Aps_5440	T/A	F: ACAAATTGAATCCAATTCTGTGGGA	FAM: ACACTGGTTAAGCTGGGGAATTTA
		R: TTCCTTTAATTCCATGAAAGTTTCCAATTTGGAATA	VIC: CACTGGTTAAGCTGGGGAATTTT
Aps_567	C/T	F: CAGTACCCCGCTTTGCATTT	FAM: CCATCTCCTTGCTGTGAACG
		R: GGTTCCTTCACAACCCTGCC	VIC: GCCATCTCCTTGCTGTGAACA
Aps_5722	G/C	F: CCCCAAAAACATCTGCTTTCTG	FAM: GATCGCAGCCACTCTACTCC
		R: GCCTCACTGCTTGCTTTGGA	VIC: GATCGCAGCCACTCTACTCG
Aps_5756	A/T	F: TGAAAAGTACTGTACATCTCCGATGA	FAM: GACCCTCTTCATTGTATTCCCAGT
		R: CCTTGATGAGGCAAGCTGACA	VIC: GACCCTCTTCATTGTATTCCCAGA
Aps_5844	T/G	F: AAGGGTACTGCCGGCT	FAM: CGGCTTAGGTTTTATATTGGCCATAGTATA
		R: ACATTCACATGCAATTTGTACCTTTTAACTCA	VIC: GGCTTAGGTTTTATATTGGCCATAGTATC
Aps_5849	T/G	F: TTCAATGTCTTGTGGTCATTAGTAATGT	FAM: AAATCTGTTAACTACCAGCCCCA

		R: CCCTCCAGTGATGTCGGGTA	VIC: AATCTGTTAACTACCAGCCCCC
Aps_6200	C/G	F: GCAGAATCGTTAGGGCCTTATTT	FAM: GATTAAGAGCGCAGACTGATTCATTAC
		R: GCTTTGTCGGTGGATGCCAT	VIC: GATTAAGAGCGCAGACTGATTCATTAG
Aps_6269	G/T	F: CCAGACTGTTTGTCACTGGTAA	FAM: AGAGGTCAAACATGCCATTTCAATC
		R: TTTATCACACTCATTATGAATATTATCTGCTTAACTGAAA	VIC: GTAAGAGGTCAAACATGCCATTTCAATA
Aps_665	G/T	F: TCCAAAGAGGGTTAAGGTATCTGT	FAM: GGGACCCGTATGAAATTGAAACAG
		R: TTTGTTTAGCGATCCATAAAAAGTGAACATGT	VIC: GGGACCCGTATGAAATTGAAACAT
Aps_6735	G/A	F: CCCTAAGACGCTCCCTTGG	FAM: ACTCCACAAGTTACCAGTTGTTCC
		R: CCTCTCCGGTAGATCCCAAAAGA	VIC: ACTCCACAAGTTACCAGTTGTTCT
Aps_7032	T/C	F: ACATTTCGACAGCGCCAC	FAM: CACAAAAGCCGAGAGAAAACCTT
		R: CCTCTATCTCCGCTCTCCCG	VIC: CACAAAAGCCGAGAGAAAACCTC
Aps_7142	G/A	F: CAATGCATAAAGGCAAGGCTC	FAM: TCACAAAGACCACTGACCCTG
		R: CCGGTCACTGGACCTAAAGACT	VIC: CTCACAAAGACCACTGACCCTA
Aps_7176	T/C	F: CACAGATGGACTATATTTATCGAGGC	FAM: CAGAGGAGCGAAGCTGTTCTAA
		R: TGCCATCTGTGGTCACCCA	VIC: CAGAGGAGCGAAGCTGTTCTAG
Aps_7795	T/A	F: GGGCCTAAACACTGCACATTC	FAM: GGAATGGCAATTTCCATGGAGT
		R: CCGGAGTCGCGTGCGT	VIC: GGAATGGCAATTTCCATGGAGA
Aps_8402	T/C	F: TCTGGTTTGTGTTCTTTCTGGATTT	FAM: TCAGAAGGTCAATGAGAAAATCCAGA
		R: GACCGATCCACCACCTCGT	VIC: CAGAAGGTCAATGAGAAAATCCAGG
Aps_8787	G/C	F: AGCTACGGCTGTACCTTACC	FAM: CAACAGGCAACTAGAAACACTTTCC
		R: AGAGCCCACTGTCATGCAGTT	VIC: CAACAGGCAACTAGAAACACTTTCG
Aps_8845	T/A	F: GCTTTCACTAGCTTCTGTCAATGTA	FAM: ATGTATACATAGCTTGTTCTAGGTCAGTTATTT
		R: CCAGCTCAGTCTCAGCAATTCC	VIC: GTATGTATACATAGCTTGTTCTAGGTCAGTTATTA
Aps_8848	T/A	F: GAACACGTCAACATTTTTTTCTGTATAAGT	FAM: CGTGCTTGAATTTTTCACCAGACA
		R: CCTATGGACTTTAATGTTTGGATTTTTTATATGGGT	VIC: CGTGCTTGAATTTTTCACCAGACT
Aps_8879	T/G	F: TGATGAATAAGAAGGTTTGGACCAC	FAM: CACTGCGATTCCTAGTTCCAGT
		R: GATGTCCAGCTGCTCTCCCT	VIC: ACTGCGATTCCTAGTTCCAGG
Aps_8962	A/G	F: GCTTTTATCGTGGTGGTCTGTT	FAM: TTGAAGTCACTGTAACACCTGGT
		R: GCTGACAGGCTACTTATAGCTTTTGC	VIC: TGAAGTCACTGTAACACCTGGC
Aps_9107	G/A	F: TGCCACAACAAATAAGAGTGTTACA	FAM: CCCAGTGATGTGCAGTAGCG
		R: CACGGTGGCCAAGACATTCT	VIC: CCCAGTGATGTGCAGTAGCA
Aps_9232	T/G	F: TGTTGATATTTCAGAATTGTAAGCTCCA	FAM: TCCATCATGTTCACTCACAGACAAA
		R: CACGTCATATAACAAGAGAAATATAAAACAAACCCA	VIC: CCATCATGTTCACTCACAGACAAC
Aps_9283	C/T	F: TTTTGGCGTTTTTGTTTGACCTT	FAM: TGTGACTTAGCCCCAGAAAGATTG
		R: GATGCACCACGCCCTGG	VIC: GTGTGACTTAGCCCCAGAAAGATTA

Aps_9493	A/G	F: CATTCCATATGTTATCTTAATGCAGAGGA	FAM: AAGTAGATTGGGGCCCAAATAGAAT
		R: CCTGGCAATAGCCCCTTTCA	VIC: AGTAGATTGGGGCCCAAATAGAAC
Aps_9518	C/T	F: CTGTGTTAAAAACGTACAGATTGAATATTGA	FAM: TCGATTGAACTGTAAGACCCATCG
		R: CCAGAGCAGCCGGGTTT	VIC: ATCGATTGAACTGTAAGACCCATCA
Aps_9753	T/G	F: CCGTGACGTACACAAATAGCC	FAM: CCGTCAAGGATGATTAATGCTTTGA
		R: ACCTGCAGCACCCTGTAAACT	VIC: CCGTCAAGGATGATTAATGCTTTGC
Aps_9757	G/A	F: CCAATCAATTCAGCTGACAACACA	FAM: ACCCATTCTATTCTGACTGAAACGTAC
		R: ACACTTCAGGGTAGCGACCAA	VIC: ACCCATTCTATTCTGACTGAAACGTAT
Aps_9847	T/C	F: TGTGTGCATAAAGAGCGCAG	FAM: CGCAGCTCAATGTCAATGAAAGA
		R: CCGGCCGCACCACATT	VIC: CGCAGCTCAATGTCAATGAAAGG
Aps_9958	C/G	F: GGACTTGATCTTTCTAATCTCCAACA	FAM: CAGCTTTTTTTAACCACACACACAC
		R: TCCACTGTTCCCATCTACTGCAA	VIC: ACAGCTTTTTTTAACCACACACACAG
Aps_9979	T/C	F: TTTTAACGCTAAGTGCCACCTC	FAM: CACAAGTTGGTGCCTGCT
		R: AGGTCTGTTTCATCTGGGGCA	VIC: CACAAGTTGGTGCCTGCC
Aps_9982	C/T	F: CATCACTCACAGCACATTGGT	FAM: TAATTTTGAAAGGCCAGTTCTACACG
		R: TGGGGATTGCTACTGTTAAATGTATTTAACTATTATGT	VIC: GTAATTTTGAAAGGCCAGTTCTACACA
Aps_1122	C/T	F: AGGTGACGTGACGTTCCTATAAG	FAM: GTTAGTGTTAACTGACGTGTTAACTGAC
		R: GCCCCTCCTGCTACTTTGAGA	VIC: AGTTAGTGTTAACTGACGTGTTAACTGAT
Aps_14730	A/T	F: GTAAATATTTACACTGTCTCCCAATGATGA	FAM: GTAAATATTTACACTGTCTCCCAATGATGAAA
		R: CACCTGGTGCTGGAGGTCT	VIC: GTAAATATTTACACTGTCTCCCAATGATGAAT
Aps_14949	G/C	F: TGGTGATTTTAAACAAGCGTATTGTTTT	FAM: TTTTAATGAGATAGGCTAATGCACAATGTTAC
		R: ACCGCCATGTCACTCACTCT	VIC: TTTTAATGAGATAGGCTAATGCACAATGTTAG
Aps_15051	G/A	F: GGAAAAGGACAGGCACCTAC	FAM: CGAACACCTACCAGAGGGATG
		R: CGTGTGGCTTCGAGCCAG	VIC: CCGAACACCTACCAGAGGGATA
Aps_15194	C/G	F: CCCTCCCTGTGATCTTTGC	FAM: CAACTTAGGAATAGGTTAATTAAGCTGTTTCC
		R: TCATGTACCTCTAAAATGTCACTTAATTCATCACAT	VIC: CAACTTAGGAATAGGTTAATTAAGCTGTTTCG
Aps_15910	T/C	F: GAGTGCATAAACACCTGCGAA	FAM: GCGAACCCAGATAACTGGTCA
		R: CGGGTGCCTTTAAATTCAGATGCAA	VIC: GCGAACCCAGATAACTGGTCG
Aps_16114	T/G	F: TGTGTTTCCCCCAGGCA	FAM: CCCCAGGCATTTACACAAAACAT
		R: TGGGCACTTAAGTGGTCCTGT	VIC: CCCCAGGCATTTACACAAAACAG
Aps_16139	C/T	F: AGATCGATCCCATCTGACTTGT	FAM: CTGTTTGTTGCCTGAGCCTG
		R: AGGCTGCCAGGAGCTCAG	VIC: TCTGTTTGTTGCCTGAGCCTA
Aps_16862	C/A	F: TGTCAGTGCAATGAAGGAAACG	FAM: GCATACAAAATTCAAGCATAGATAACATTTCAAAC
		R: TTGCTGACAGCCAAGAATTGATTTGT	VIC: GCATACAAAATTCAAGCATAGATAACATTTCAAAA
Aps_1838	G/C	F: CCGTTTTCCGTTAATAGGGACTTT	FAM: CCCCATCACTAACCCACATCAC

		R: GGGTTCCATGCACACCTGAT	VIC: CCCCATCACTAACCCACATCAG
Aps_19806	A/G	F: GCAGTTGTCCAATATGGTCAATCT	FAM: GTCTCAATTGTGTTTCGTGACGT
		R: AATTCAAGTGCATGAATGTGTAGCTTCTG	VIC: TCTCAATTGTGTTTCGTGACGC
Aps_21003	A/T	F: GTAGTGTGTCAGGCATTGTACT	FAM: ACTGTCAAATGGGTCATGCTGAA
		R: GATATGTAACTAAAACGATCAAACATGGTTAGATTTTCC	VIC: ACTGTCAAATGGGTCATGCTGAT
Aps_2109	T/G	F: CCTAGCTATTGTGTGTGTGTGT	FAM: TCTTGTGTTCATTTCATGCACTCATTT
		R: GGTGAAGATGACCTCAAAGATAAGTCTCA	VIC: TCTTGTGTTCATTTCATGCACTCATTG
Aps_22011	T/C	F: GGATAAGTTGAGGACACCTATTTGT	FAM: GGATAAGTTGAGGACACCTATTTGTCTT
		R: CTCTCCACTTCCCAAATCGCAT	VIC: GGATAAGTTGAGGACACCTATTTGTCTC
Aps_22309	G/A	F: ACCAGGTACAGGCTTAGTGAC	FAM: ACCTAGCAATAGAAATGGGAAGACG
		R: TGTTCTCTTGGCAGCCAGGT	VIC: AACCTAGCAATAGAAATGGGAAGACA
Aps_2579	C/T	F: AGATCAGAGGAACAGCAAGGT	FAM: GTCATAATAACAACGAGAGTGGAGAAAATC
		R: CCCTAGTGCAGTTTACAAAAAGTCCTTTAA	VIC: GTCATAATAACAACGAGAGTGGAGAAAATT
Aps_25940	C/A	F: GTGTATGAAGCACCTTTCAACTATGA	FAM: AGAAAATTGAGTTTGCTGTCATACAGTTG
		R: ACCAGGGATCAACCACAACTTTCA	VIC: AGAAAATTGAGTTTGCTGTCATACAGTTT
Aps_3073	A/G	F: AGGGAGTGTGCCTGTGT	FAM: CCTGTGTTTTATCAGAATAAAAATTAAACACTCCT
		R: CGTGGCAGAGACACTTGGG	VIC: CTGTGTTTTATCAGAATAAAAATTAAACACTCCC
Aps_3266	G/A	F: GCGCCTTTATAAAACAAGACCTG	FAM: CGCCTTTATAAAACAAGACCTGAAACG
		R: CCCTATGATCCAGCCGAGGG	VIC: CGCCTTTATAAAACAAGACCTGAAACA
Aps_686	A/G	F: TGCTATGACAATAGTATGGTGGACA	FAM: GACAAAAATCTATGGCAGACTTTACCT
		R: ACCATTATAGAGCTGCTTTTGTACAACCA	VIC: GACAAAAATCTATGGCAGACTTTACCC
