# Supplementary material for: Discovery and characterization of single nucleotide polymorphisms in two anadromous alosine fishes of conservation concern
Source: Ecol Evol. 2017 Jul 18;7(17):6638–48. doi: 10.1002/ece3.3215 (PMC5587496; doi:10.1002/ece3.3215)
Supplement: Supplementary file 4 [file ECE3-7-6638-s004.pdf]

Table S3. Details of all SNP genotying assays for blueback herring

Assay name	Assay target	Primers (5'-3')	Probes (5'-3')	GenBank No.
Aae_10384	G/T	F: GTCTTGGCTCAAATAATGTTTCAGG	FAM: GCCTCAGGTCATCATCAGGTTC	
		R: CATGTGGCATATCAGAAAAGCATGGA	VIC: GCCTCAGGTCATCATCAGGTTA	
Aae_10678	A/T	F: TTTCTTGAGACAGGCCCTTTTG	FAM: CCTTTTGAGTTTTGGAAAACACTGGTA	
		R: GTTTAAAACTATTTCATTCAAGTTGACTGATCATCTCA	VIC: CCTTTTGAGTTTTGGAAAACACTGGTT	
Aae_10801	A/G	F: ACATGCTTAAAATATTGTGTTGGTACCT	FAM: GTGTTGGTACCTTGACTCAAACTATTTT	
		R: CACCCTCAGCCATGAAAACCAT	VIC: GTGTTGGTACCTTGACTCAAACTATTTC	
Aae_1107	C/A	F: ACTTTAGCCTACTTCAGTACAGATCA	FAM: AGATTAATATGTTCAATAATGTCTAGTCAGTACACC	
		R: ACATATTTTATTTTCACAATTTCTATATAGGCCTTCCTTA	VIC: AGATTAATATGTTCAATAATGTCTAGTCAGTACACA	
Aae_1144	A/C	F: GGTTTTGACAGGGCCATCTAT	FAM: TGTCTTTTCATGGTTAAAGTAGCACAAAATT	
		R: ACACATATACTTACATATACCTACCAAGTACGTCA	VIC: GTCTTTTCATGGTTAAAGTAGCACAAAATG	
Aae_1275	T/A	F: TTTGTTGAATTGCGGTCCTGA	FAM: GTCCTGAAGATGTATGAGTAGCCTT	
		R: GACGTGACCAAATCCCGCTG	VIC: GGTCCTGAAGATGTATGAGTAGCCTA	
Aae_1287	C/G	F: GGCCTAGCGTCCCTAGT	FAM: GTCCATTCAGTTGAATAGACATCCAAAC	
		R: TTGATCTTTTTACATTATAGCGAAATACAATATGCGTC	VIC: GTCCATTCAGTTGAATAGACATCCAAAG	
Aae_1354	T/C	F: CACACCCTTGCTGTCTCAC	FAM: CATAGGCCTACTCTGCCAGTT	
		R: GCATTTTTGTAAAATGTGACCTATTTTTGAATGTGA	VIC: CATAGGCCTACTCTGCCAGTC	
Aae_136	C/T	F: TTAGCATCTAACCAGAAGTGCAAAA	FAM: GCAGAAAAGTGCAATGTCAACAGTATAG	
		R: CCCTCTTCACCTACTGCACACT	VIC: GCAGAAAAGTGCAATGTCAACAGTATAA	
Aae_1454	T/G	F: CCTCTCCTGGCTGGCT	FAM: AAATGTCGTGGTGGAAGGTCATA	
		R: TGAGCTAAATTTTCTACTTATCTACTGGCCG	VIC: TGTCGTGGTGGAAGGTCATC	
Aae_1482	G/A	F: TTGAAGGAACAGTCCAGAGGA	FAM: GGTTTAACAAACTAACAGTCTAACCTCG	
		R: CCATCTCATCTCAGGGTAAATCAACTCA	VIC: AGGTTTAACAAACTAACAGTCTAACCTCA	
Aae_1504	C/A	F: GCCTGTCGCCAGCAAC	FAM: GCTCTGGTGCGCATTTAGTC	
		R: GAATCTCGGGATAGGGCCGAA	VIC: TGCTCTGGTGCGCATTTAGTA	
Aae_1605	C/G	F: GAGATACATTTTCGATCAATGTGTAGGT	FAM: TTAATTGTGTAGGGTAATTAATTTAACAGTATAAAC	
		R: ACTGGTATTCCATTTTAATATCAGCCTACAAGA	VIC: TTAATTGTGTAGGGTAATTAATTTAACAGTATAAAG	
Aae_1792	C/A	F: CGCACAGTTAGCACTGACAAC	FAM: CCCGGCAAAAGCTAATTACCAC	
		R: CCCTGCCAGGTCAGTGAGT	VIC: CCCGGCAAAAGCTAATTACCAA	
Aae_2	G/C	F: GCAGCGGGAAGCTGTT	FAM: GTTACGTGACTGACCAACACAAAG	

		R: ACATGGCCTCCTACCATCCAA	VIC: GTTACGTGACTGACCAACACAAAC
Aae_2120	C/A	F: GCATTTGCGGTGGTCTCT	FAM: GAGAATACCTAGAGTATTCGTCATATCCTG
		R: TGGGTGTTCTCACAGCAGCTA	VIC: TGAGAATACCTAGAGTATTCGTCATATCCTT
Aae_2136	C/T	F: TCCTTTTACAGAGATTGGAGAGGG	FAM: GAGAAGAAACCTGCTGATAAATCATTCTTTATC
		R: GGTCCGTGCTCTCTGAAGGA	VIC: GAGAAGAAACCTGCTGATAAATCATTCTTTATT
Aae_2146	G/A	F: CATGTGTTTTGTTGTTTTTGGAGAGA	FAM: AGGACGGGTGTTGTTGACTC
		R: GGTTGACTTCCTCCTAAAGTCTTCAATATGA	VIC: AGGACGGGTGTTGTTGACTT
Aae_219	G/A	F: AGGCTGCACTGTGGGT	FAM: TGGTGTGCACAAACACAACG
		R: AGTCTCCACAGCTCACCACG	VIC: CTGGTGTGCACAAACACAACA
Aae_2394	A/C	F: CTACAACCCCTACGGTTTCAATC	FAM: CGGGGCAGTTTCAGGCTT
		R: CTGTGTTGGCCAGGACAACT	VIC: GGGGCAGTTTCAGGCTG
Aae_2420	C/T	F: TGTCGGCCTCGCTGTAG	FAM: TCCCATCCAGCATCATGGTC
		R: AGGCCCTACCATGTAGCTGTG	VIC: TCCCATCCAGCATCATGGTT
Aae_2483	G/T	F: GTGAGCAGTATGACATCAAGACAT	FAM: GATTACTGTACAGCACTTTAAAAGGAAAAATG
		R: GGTGTTGGTGATCTGATCTTCATTTTTAAAGT	VIC: TGATTACTGTACAGCACTTTAAAAGGAAAAATT
Aae_258	G/A	F: CTCAATGAACCCCAGAACCTT	FAM: TGAAAAACCTGCTCCAACATGC
		R: TCATCATCAGAATCATCTAATATATCTTTATCTTCAACTT	VIC: CTTGAAAAACCTGCTCCAACATGT
Aae_2650	G/A	F: AAACCCATTATTTTCATTGTTGGTATTTGA	FAM: GATATCGTTCTTATCATTGTTGTTAGATGACTAATG
		R: CCCGCTGTCTTGCCGT	VIC: GATATCGTTCTTATCATTGTTGTTAGATGACTAATA
Aae_2668	T/C	F: ACTTCGCTGTGCCCTTCA	FAM: GCCCTTCAGCTTAGATTTAACTGGA
		R: GAGTGCACTCGCATTGTGGT	VIC: CCCTTCAGCTTAGATTTAACTGGG
Aae_2726	G/C	F: TCTTATCGCTGGCTTAATTGTTTCT	FAM: TCAGTGCCAATGGCTTTGC
		R: TGCGTGACAGATGCTTGGTGA	VIC: CTCAGTGCCAATGGCTTTGG
Aae_2937	A/G	F: CACCTTTTACATTTTGTTGGAAGCT	FAM: GCTGGAAACTCGGTAATGCTCATA
		R: TCCCTCCACTTTGGCTGACTT	VIC: CTGGAAACTCGGTAATGCTCATG
Aae_2984	A/G	F: GTTCCTAAGCAACACAAATGGTTT	FAM: GCCTAAACTGATGATTGAATACGCTT
		R: GTGGCAGCACTGGCGT	VIC: GCCTAAACTGATGATTGAATACGCTC
Aae_304	G/A	F: GGCTAGTCAGGAGTGTGCT	FAM: GTGCTGATCTTTGGCTCAGTTG
		R: CAGTCACCACTCCAAGGAGTCA	VIC: TGTGCTGATCTTTGGCTCAGTTA
Aae_3391	A/T	F: ACAAAAGACACACACAGCACA	FAM: CACAAAGACTGTACACAAGAAACCA
		R: TCGTCTCAAGCACAGCCACT	VIC: ACACAAAGACTGTACACAAGAAACCT
Aae_3432	A/C	F: GGGAACGAGTCAGAATGCAA	FAM: CACAACACGCACCATACAGAA
		R: TCACCAAGCCCTGGAAGCTC	VIC: CACAACACGCACCATACAGAC

Aae_3704	C/G	F: TCCTCACGTCACTCTTCACATAC	FAM: CTGTGGAAAGTGTAAAGACATACTTGC
		R: TTTATAGATAGAACAATTTGGACATTTTTGTCAAGTAATG	VIC: CTGTGGAAAGTGTAAAGACATACTTGG
Aae_3706	A/G	F: GTCACACTCGCATGGGC	FAM: CTTCCGTGTGTTAAGTGTACCACTA
		R: GTCACACCTGCAACACACCC	VIC: TCCGTGTGTTAAGTGTACCACTG
Aae_3749	A/G	F: CCCAAGCACCTTGTACGG	FAM: GATCAAAAGGAGGGACTGATTCCA
		R: GCCAGCTGTGGTCATCTGTG	VIC: TCAAAAGGAGGGACTGATTCCG
Aae_3768	T/G	F: ATGATCATGTCAGTGTTTTGAACGA	FAM: TGAACGAATCTAACTAAAGAATGGAAGCA
		R: GTGCCTGAAGGCTCTTCATGG	VIC: GAACGAATCTAACTAAAGAATGGAAGCC
Aae_3882	G/T	F: TCCAGTATCTACAAAAGCATTACACAAA	FAM: AGCATTACACAAACATAAAAGGTTATACAGTATTC
		R: ACATCTGACACAAGTTGATATAATGAACTGAATATGA	VIC: AAGCATTACACAAACATAAAAGGTTATACAGTATTA
Aae_3942	A/T	F: GTAGGCCCGTTCAGTAATCC	FAM: CATCCCTGCTTGGACTGAAGA
		R: AGCGGCCTGTGAAAGGCTAT	VIC: CATCCCTGCTTGGACTGAAGT
Aae_4063	A/T	F: GCCCTTACATGCCTCCATTT	FAM: GGTTTTCATCACCAAAGGAGACCTA
		R: GCTGGTTCTGTTTCTGCGGT	VIC: GTTTTCATCACCAAAGGAGACCTT
Aae_4154	C/A	F: CAGAACGCTGTCAAGGAGC	FAM: GGTCACTTCTTGCAATGGAACTTC
		R: ACACAGCTCAACCGTCTGCT	VIC: TGGTCACTTCTTGCAATGGAACTTA
Aae_4191	C/T	F: GTGACATCTCTCCCCGCT	FAM: CGTCTATTTTGGGAGGCGAAAAC
		R: AGGGCAGGCTAATGTGAGGAG	VIC: CGTCTATTTTGGGAGGCGAAAAT
Aae_4223	T/C	F: GTTGCAAGTAAGTCAAATATCAGCC	FAM: CAAGAGTATACCCTACAACAGCAGT
		R: GGGCTTTATGTAGGCCCACTG	VIC: AAGAGTATACCCTACAACAGCAGC
Aae_4247	C/A	F: CTGACCGGTTTGCCCC	FAM: GCACCTCCAACCCCATGG
		R: GTATGCGGCACCTTGGCATT	VIC: GCACCTCCAACCCCATGT
Aae_4287	G/C	F: CAGGAGTGCGGATACATAAAGAC	FAM: GCACACTTACACCCCTCCG
		R: GTGTTTGCCTGTCTATGTGTGTGT	VIC: GCACACTTACACCCCTCCC
Aae_430	G/A	F: CTCAGCTACAACTGGGACATTT	FAM: GAATGAGTATTGCAGCTCAGCG
		R: ACACACACACACCTACATATTATGAGCA	VIC: GGAATGAGTATTGCAGCTCAGCA
Aae_4366	T/C	F: AAAGCAACAAGATTCGTTCTTCTCT	FAM: CTAATCTGGTCTCAGCATGGGTA
		R: GTCCAGGAATAGCCTTCTGGGTA	VIC: ATCTGGTCTCAGCATGGGTG
Aae_4389	G/A	F: CACTTTGTCCTAGAAATAACACCGA	FAM: AAGAGCTAATGCTTTCAGCTGATATTTG
		R: GCAAGACAATTTAATTTACATATAGGCCTAATGTCA	VIC: GAAAGAGCTAATGCTTTCAGCTGATATTTA
Aae_4472	A/G	F: CTTCACCAGTTCCTACGAGAAG	FAM: AAGAGGCCTTTCGATTGGCTA
		R: TGTTCGTCCTCCTCCTTGAGC	VIC: AGAGGCCTTTCGATTGGCTG
Aae_459	G/A	F: CGGACATGACAGTGTTTTTATTGC	FAM: GCAATGTACAGATGGTCTGCG

		R: ACGTATCAAACACTTTCAGTCAATAAAATAGAGATCAA	VIC: TGCAATGTACAGATGGTCTGCA
Aae_4602	G/T	F: TGTTGACAGCAAAATCTAGCACC	FAM: GCACCCTAACTCCACTCCAC
		R: CAGTGGATACAATGAATGAACCATTGTGTT	VIC: GCACCCTAACTCCACTCCAA
Aae_4733	A/G	F: TGGAAGCCAGCTAAGGACA	FAM: CATACATTTGCCAACACAGGATAGAA
		R: CGGTAAATATCTAAATTCTTTGCAAGTCAAAGGAA	VIC: CATACATTTGCCAACACAGGATAGAG
Aae_4940	T/C	F: CCCGAGAATTTGTAATACATAAGTGGA	FAM: CTTCATGTCAGGTCAGAGACTGTA
		R: CCCCTTTCCTCATTTTCCCCC	VIC: TTCATGTCAGGTCAGAGACTGTG
Aae_4985	C/T	F: TGTTTTCTGATTAATTGCAGTTGCG	FAM: CGGCTCCTCTAGGTTGGG
		R: CACCTGAACAGGTTACCTCCGA	VIC: CGGCTCCTCTAGGTTGGA
Aae_5113	C/G	F: CGACCATTAATCGTAATTGCTGACT	FAM: TTTCTTTGTATTGGTTGCCCTGC
		R: GGAGTATTCCACCCAGGGCT	VIC: ATTTCTTTGTATTGGTTGCCCTGG
Aae_5177	A/C	F: CAACGTGCCGCTGGAT	FAM: AAGGATCAGCAGAACATGTCGT
		R: CCCAACCCAGCAACTATGCAG	VIC: AGGATCAGCAGAACATGTCGG
Aae_5262	A/C	F: CCTTTCATTTAGAGCTGACAATTGG	FAM: AGGTTTAAAGCACAAGACCAAATTAACAT
		R: CGCTGCTCCATCACACCC	VIC: AGGTTTAAAGCACAAGACCAAATTAACAG
Aae_5492	A/G	F: TCTTTGTTTCTAACTCACTTGCCC	FAM: TTTCTAACTCACTTGCCCTCTTTCTA
		R: ACATACAGACAGAGAGAGAAAGTATTAGCGT	VIC: TCTAACTCACTTGCCCTCTTTCTG
Aae_554	T/C	F: TGCTAGCTTAAATGGGAGTACACTA	FAM: CAGCATACCTTTGTGAGCTCTACA
		R: CACAGCAGAAGTGTGCCGA	VIC: AGCATACCTTTGTGAGCTCTACG
Aae_5563	T/C	F: GTGCCGTGGATTACAAGGT	FAM: GTTTACAAACCCCTACCACCTTTT
		R: TGTTGCCTTTATCCTCCATATCTTAAATTGACT	VIC: GTTTACAAACCCCTACCACCTTTC
Aae_5703	G/C	F: TGCCTTTGCAATTATCACACTATGA	FAM: ACCAGAGATTCATGTATACACATCTTGTC
		R: ACACACAAGTACAGATGCAGGGT	VIC: ACCAGAGATTCATGTATACACATCTTGTG
Aae_5737	T/A	F: AGGACTGAGGAGCTCTTTGT	FAM: GTCCAACACCGAGCAGAAGT
		R: AGTCGATTTCCTGGCTGCTCT	VIC: GTCCAACACCGAGCAGAAGA
Aae_5826	A/G	F: TGGTGTCAGATTACAGATCTCCA	FAM: GGCGACTGAACTCCCGT
		R: ACGCACATCCATCTCCACACA	VIC: GGCGACTGAACTCCCGC
Aae_5833	C/A	F: CAGAGTTCTAGAAATCTGATGGGC	FAM: GGGCATTATGCTAGACCTCTAGTTG
		R: CCTCAGAACACAAAGGCCACAA	VIC: GGGCATTATGCTAGACCTCTAGTTT
Aae_5886	A/T	F: CCTGCAAGTGTAAGTGTCCG	FAM: GCCTTACGGCTGTATAACTGGT
		R: CCTGGGCATTGGTGAAAGGTT	VIC: GCCTTACGGCTGTATAACTGGA
Aae_6021	A/T	F: CCAATTCCAGTTTTGCATCTTTGA	FAM: TGCTGTTGTTTTGCTTTTGGCT
		R: GGCTCCACACACAAAAGCCAT	VIC: TGCTGTTGTTTTGCTTTTGGCA

Aae_6083	C/G	F: GGAACAGAATGGAATACTAAGGCA	FAM: GCATCTTTCTTCTATCCAAAGTGCG
		R: GTGTCCAAGATATGGATATGATTTTCAGCTTT	VIC: GCATCTTTCTTCTATCCAAAGTGCC
Aae_6273	C/T	F: GGAGCAACTGATATGCCTTTACA	FAM: AATTGAAAGGTTCACAGTTCTGCTG
		R: AGCCGTGTTCACGCTCTCA	VIC: CAATTGAAAGGTTCACAGTTCTGCTA
Aae_6302	C/T	F: CAGCTTGGGTAATGTAGTTAATTGC	FAM: TTTACATTGGGAATGGATTGCCG
		R: GCCCAATCCACAAAACAGTGAGT	VIC: CTTTACATTGGGAATGGATTGCCA
Aae_6327	A/G	F: TGTTAGATTTGATGACATGACGCC	FAM: CCTTGTCAGGCCTCCTTGT
		R: GCATCACATTCTGTAGGGATTATTTCAAAGT	VIC: CCTTGTCAGGCCTCCTTGC
Aae_6571	A/T	F: GAAGCTATCCAGCCAGCC	FAM: CCAGCCAGATACAGCATGGTTT
		R: GGCTGACTGTTTCGACATGCAA	VIC: CCAGCCAGATACAGCATGGTTA
Aae_6703	T/C	F: CTGCTTCAAGGTTCAAGTGTTACA	FAM: CGTTCAGAGATACTCCTCTGCAT
		R: GCTGATGGAAAGGCAACCGT	VIC: CGTTCAGAGATACTCCTCTGCAC
Aae_694	C/T	F: TCCTGGGCTAACCCCC	FAM: CGCCACCTTGTCGTGTCC
		R: GGAAAGGAGCTGTAGCCTGACA	VIC: CGCCACCTTGTCGTGTCT
Aae_7011	T/A	F: CTGTGTGTTTGTGTGTACAGGTAT	FAM: TTTGTAGGGTTTCATAGTGTGCTAGT
		R: GCGAGCGCTTCCTAAATATTTTCAAGA	VIC: TTTTGTAGGGTTTCATAGTGTGCTAGA
Aae_7040	C/T	F: GAGCGCAGACTGATTCATTAGAG	FAM: CCAGGAAATGGCATCCACC
		R: GCTCACGGTGGCTGTTCTG	VIC: ACCAGGAAATGGCATCCACT
Aae_7090	A/G	F: GGCGTGTGCAGTCCAA	FAM: AATTGCCATCCTAACGGGGTAATA
		R: CCCTTTTGAACAGTGTTCCAGGC	VIC: TGCCATCCTAACGGGGTAATG
Aae_7200	G/T	F: TGTTTGTTTTTCATTAAATCCCACTCAAA	FAM: AATCCCACTCAAACTTTAATAAATTAACCAGAG
		R: CACACACACACACACAATAGCTAGG	VIC: AAATCCCACTCAAACTTTAATAAATTAACCAGAT
Aae_7269	C/A	F: GGATCTGTTTTAGCGTCGCTATAT	FAM: CATGGTATCTTTTGCTCGCAACG
		R: GAGGTTGATGGAATACCCCCCA	VIC: CATGGTATCTTTTGCTCGCAACT
Aae_7796	C/T	F: CCTGGGTATTGTTGTTCTGGC	FAM: CACCTTCCCTTTGATATTGGACAAC
		R: TGCCTACTGCTCGTTAAAGCTCT	VIC: CACCTTCCCTTTGATATTGGACAAT
Aae_8427	C/T	F: GTGAGGGAGAACTTCATTACTTCC	FAM: CCTGGACATGTTTTGGCCTC
		R: GCAAGCCAGTTCTCCTCCATT	VIC: TCCTGGACATGTTTTGGCCTT
Aae_8435	C/T	F: GGACAAACTCAACAACCAGTGAAA	FAM: ACAGTAAATACCATCTGAGTCACTGTTAC
		R: ATCAGCTAGCTGGACCCAATGT	VIC: AACAGTAAATACCATCTGAGTCACTGTTAT
Aae_8734	A/C	F: CTTGCAGCTGATGTGTACAGAA	FAM: AGTTTTGGTGGGTTTTTAAAAGTGTATTATTATTAT
		R: AGTCTGTTATCATACATCTTTTTTTCAACTGAGTTTATAT	VIC: AGTTTTGGTGGGTTTTTAAAAGTGTATTATTATTAG
Aae_9052	A/T	F: TGTGACTGCTCTTGTTGGC	FAM: TGGCTAATTGCCTTAACCAGGAA

		R: TCGACTATTAAAATAATTAGTTGTAGCCGTAAATAAAAAT	VIC: TGGCTAATTGCCTTAACCAGGAT
Aae_9057	C/T	F: GGGCATTTTCACATCAGCTACT	FAM: CACTCAGATACAATGAAATGCGTACC
		R: AGGCTGACTACAGCCTGTGTT	VIC: CCACTCAGATACAATGAAATGCGTACT
Aae_928	G/T	F: TCAGCAAATGAACAGTGCAAGT	FAM: AGTGTTTCAGACCTGTGAAATGAAATG
		R: CATGACATTTCCACGAATAATTAATAATCACGGA	VIC: AAGTGTTTCAGACCTGTGAAATGAAATT
Aae_11731	T/C	F: GGAGTAGAGAGACATTCTACTGCT	FAM: CTTCCCGCTAAGGATGAGCA
		R: CTGCCATTAATTTACACAGCAGAATATGGT	VIC: TTCCCGCTAAGGATGAGCG
Aae_11754	C/A	F: ACCAGGAATACATTTGGCCATTTT	FAM: TGGTGTGACCTACAACAGACC
		R: CCACTGTCATATATACCACATAGATTTTAGACCA	VIC: TTTGGTGTGACCTACAACAGACA
Aae_3449	T/A	F: CAATGCCTATATCTCTGACATGTCAA	FAM: TCAAATAATAAGGAGATGCCAGTCCATT
		R: GGATTGTGCTTTGATATTTATAGTACTATCAATAGAACTT	VIC: TCAAATAATAAGGAGATGCCAGTCCATA
Aae_462	C/A	F: GCTGCATCTACAGGGCATCT	FAM: TTGCATGTTATGCTGCATGTACTATTC
		R: GCATCTCTCTGACTTTAAAGACTAATCCTTCA	VIC: CTTTGCATGTTATGCTGCATGTACTATTA
Aae_6727	G/A	F: ACTTCAGTGGATATCTGGACAACA	FAM: ACAACACAGAGTGCTTTTGACAAAATATAG
		R: GACTTGATTTGACTCTGTCAGGTAGATAACT	VIC: ACAACACAGAGTGCTTTTGACAAAATATAA
Aae_6831	A/G	F: GTTTACTTCAATCAAATGAGCGAGC	FAM: CGAGCATAGGCTGCTGGATT
		R: ACAAACCAGACATTTATGTATTGGATGACAATG	VIC: CGAGCATAGGCTGCTGGATC
Aae_71	C/T	F: CCTTGGGTTTGGAGCTTGC	FAM: GAGCACCAAGGAAACGGC
		R: CCAGGCGAATGACTCGTAGGA	VIC: TGAGCACCAAGGAAACGGT
Aae_8252	G/A	F: ATGGTTAACAAACTTGTAATAGGGCA	FAM: GCATAGCTGATTAGCGACCGATC
		R: TGACGAGACCTGAAGCTGCAA	VIC: GCATAGCTGATTAGCGACCGATT
Aae_2367	T/G	F: TGCACATATTCAGTATCAATGCAGG	FAM: AGTATCAATGCAGGGGCATACA
		R: ACACGATTTAAAGTCAAAGATGATCCTCTCT	VIC: AGTATCAATGCAGGGGCATACC
Aae_4025	C/T	F: TGTTAAAAAGAGAGCCTTGCATCA	FAM: AAACATCAAAAAGAGAGCCCTGC
		R: CAGGTTCAACACAACGCAGGT	VIC: AAAAACATCAAAAAGAGAGCCCTGT
Aae_5780	A/T	F: ACGACTTCACATGAATGAATGAATGAAT	FAM: AATGAATGAATGCACATTCACAGGT
		R: GTGAGTCTCACGGCCAATGC	VIC: GAATGAATGAATGCACATTCACAGGA
Aae_5869	C/G	F: GGCCATTATGGCAGTCAATCTT	FAM: CTTAATCTGTGACCAAAACATTTAAATCGG
		R: CATTTTTACATTTACATTTATTAATTCAACAGACGTATCA	VIC: CTTAATCTGTGACCAAAACATTTAAATCGC
Aae_5919	T/C	F: AAGAGTTCAAATCATTCTACTGAAAGCA	FAM: GCAGTAGCTGTCTGGATGAACTT
		R: GGTTTGTGGTGTTGTTGTTCCATCT	VIC: GCAGTAGCTGTCTGGATGAACTC
