# Supplementary material for: Discovery and characterization of single nucleotide polymorphisms in two anadromous alosine fishes of conservation concern
Source: Ecol Evol. 2017 Jul 18;7(17):6638–48. doi: 10.1002/ece3.3215 (PMC5587496; doi:10.1002/ece3.3215)
Supplement: Supplementary file 5 [file ECE3-7-6638-s005.pdf]

Table S4. Alewife allele frequencies, expected, and observed heterozygosities by locus. Allele frequencies are standardized to the minor allele in the Quinnipiac River.
*** = out of Hardy Weinberg equlibrium**

	Waughs N = 27			Tusket N = 45			Androscoggin N = 47			Penobscot N = 44			Mashpee N = 40			Quinnipiac N = 36			Chowan N = 41			Alligator N = 43		
Assay name	Allele Freq.	He	Ho	Allele Freq.	He	Ho	Allele Freq.	He	Ho	Allele Freq.	He	Ho	Allele Freq.	He	Ho	Allele Freq.	He	Ho	Allele Freq.	He	Ho	Allele Freq.	He	Ho
Aps_139	0.111	0.201	0.148	0.011	0.022	0.022	0.096	0.175	0.191	0.216	0.342	0.386	0.138	0.240	0.275	0.071	0.135	0.143	0.088	0.162	0.125	0.081	0.151	0.163
Aps_257	0.148	0.257	0.296	0.133	0.234	0.222	0.141	0.245	0.283	0.080	0.148	0.159	0.063	0.119	0.075	0.042	0.081	0.083	0.085	0.158	0.122	0.058	0.111	0.116
Aps_290	0.037	0.073	0.074	0.011	0.022	0.022	0.074	0.139	0.149	0.045	0.088	0.091	0.282	0.410	0.410	0.176	0.295	0.294	0.171	0.287	0.244	0.298	0.423	0.357
Aps_521	0.222	0.352	0.370	0.131	0.230	0.262	0.300	0.425	0.422	0.244	0.373	0.390	0.163	0.276	0.225	0.319	0.441	0.361	0.338	0.453	0.425	0.360	0.466	0.535
Aps_567	0.074	0.140	0.148	0.159	0.271	0.273	0.053	0.102	0.106	0.239	0.368	0.341	0.138	0.240	0.225	0.056	0.106	0.111	0.037	0.071	0.073	0.105	0.190	0.116*
Aps_665	0.000	0.000	0.000	0.000	0.000	0.000	0.032	0.062	0.064	0.011	0.023	0.023	0.000	0.000	0.000	0.014	0.028	0.028	0.000	0.000	0.000	0.000	0.000	0.000
Aps_686	0.481	0.509	0.444	0.341	0.455	0.455	0.032	0.062	0.064	0.057	0.108	0.114	0.300	0.425	0.500	0.243	0.373	0.371	0.525	0.505	0.550	0.440	0.499	0.500
Aps_1000	0.130	0.232	0.174	0.056	0.106	0.067	0.000	0.000	0.000	0.026	0.051	0.051	0.050	0.096	0.100	0.000	0.000	0.000	0.037	0.071	0.024	0.000	0.000	0.000
Aps_1122	0.204	0.331	0.259	0.286	0.413	0.429	0.500	0.505	0.489	0.500	0.506	0.455	0.269	0.399	0.436	0.361	0.468	0.611	0.061	0.116	0.122	0.047	0.090	0.093
Aps_1475	0.000	0.000	0.000	0.000	0.000	0.000	0.032	0.062	0.064	0.045	0.088	0.091	0.025	0.049	0.050	0.014	0.028	0.028	0.061	0.116	0.122	0.012	0.023	0.023
Aps_1607	-	-	-	-	-	-	0.202	0.326	0.319	0.302	0.427	0.465	0.263	0.392	0.225*	0.097	0.178	0.194	0.167	0.282	0.222	0.110	0.198	0.220
Aps_1667	0.077	0.145	0.154	0.036	0.070	0.071	0.032	0.062	0.064	0.070	0.131	0.140	0.000	0.000	0.000	0.000	0.000	0.000	0.000	0.000	0.000	0.000	0.000	0.000
Aps_1762	0.056	0.107	0.111	0.044	0.086	0.089	0.223	0.351	0.277	0.182	0.301	0.318	0.075	0.141	0.150	0.167	0.282	0.222	0.024	0.048	0.049	0.035	0.068	0.070
Aps_1838	0.130	0.230	0.185	0.092	0.169	0.184	0.245	0.374	0.404	0.267	0.396	0.395	0.196	0.322	0.391	0.191	0.314	0.324	0.026	0.051	0.051	0.107	0.194	0.214
Aps_1929	0.000	0.000	0.000	0.044	0.086	0.089	0.000	0.000	0.000	0.011	0.023	0.023	0.025	0.049	0.050	0.000	0.000	0.000	0.061	0.116	0.073	0.023	0.046	0.047
Aps_1991	0.327	0.449	0.423	0.378	0.475	0.578	0.766	0.362	0.340	0.705	0.421	0.409	0.438	0.498	0.575	0.444	0.501	0.611	0.421	0.494	0.579	0.535	0.503	0.419
Aps_2109	0.148	0.257	0.222	0.056	0.106	0.111	0.096	0.175	0.191	0.080	0.148	0.159	0.125	0.222	0.250	0.069	0.131	0.139	0.244	0.373	0.341	0.163	0.276	0.326
Aps_2158	0.019	0.037	0.037	0.022	0.044	0.044	0.000	0.000	0.000	0.023	0.045	0.045	0.013	0.025	0.025	0.000	0.000	0.000	0.000	0.000	0.000	0.000	0.000	0.000
Aps_2556	0.111	0.201	0.148	0.133	0.234	0.267	0.032	0.062	0.064	0.091	0.167	0.182	0.188	0.309	0.375	0.097	0.178	0.194	0.049	0.094	0.098	0.093	0.171	0.186
Aps_2579	0.352	0.465	0.333	0.523	0.505	0.500	0.479	0.504	0.489	0.443	0.499	0.477	0.449	0.501	0.487	0.429	0.497	0.686	0.659	0.455	0.488	0.607	0.483	0.452
Aps_2866	0.037	0.073	0.074	0.067	0.126	0.133	0.043	0.082	0.085	0.057	0.108	0.114	0.088	0.162	0.175	0.111	0.200	0.167	0.061	0.116	0.122	0.047	0.090	0.093
Aps_2925	0.077	0.145	0.154	0.233	0.362	0.467	0.185	0.305	0.239	0.080	0.148	0.159	0.063	0.119	0.125	0.069	0.131	0.083	0.146	0.253	0.244	0.244	0.373	0.349
Aps_3073	0.093	0.171	0.185	0.411	0.490	0.511	0.287	0.414	0.447	0.330	0.447	0.386	0.191	0.314	0.206	0.194	0.318	0.278	0.197	0.321	0.395	0.262	0.391	0.238*
Aps_3089	0.019	0.037	0.037	0.222	0.350	0.400	0.096	0.175	0.191	0.182	0.301	0.227	0.163	0.276	0.225	0.167	0.282	0.333	0.256	0.386	0.415	0.233	0.361	0.326
Aps_3266	0.327	0.449	0.577	0.144	0.250	0.244	0.383	0.478	0.468	0.182	0.301	0.273	0.375	0.475	0.600	0.347	0.460	0.306*	0.162	0.275	0.216	0.233	0.361	0.465
Aps_3269	0.556	0.503	0.593	0.333	0.449	0.400	0.663	0.452	0.488	0.679	0.441	0.436	0.538	0.503	0.513	0.486	0.507	0.583	0.275	0.404	0.500	0.337	0.452	0.442
Aps_3344	0.231	0.362	0.385	0.189	0.310	0.244	0.351	0.461	0.362	0.395	0.484	0.465	0.350	0.461	0.400	0.417	0.493	0.500	0.098	0.178	0.195	0.128	0.226	0.256
Aps_3356	0.315	0.440	0.481	0.400	0.485	0.400	0.261	0.390	0.391	0.267	0.396	0.488	0.263	0.392	0.425	0.338	0.454	0.441	0.463	0.503	0.525	0.360	0.466	0.488
Aps_3404	0.019	0.037	0.037	0.078	0.145	0.156	0.096	0.175	0.191	0.159	0.271	0.227	0.088	0.162	0.175	0.028	0.055	0.056	0.012	0.024	0.024	0.000	0.000	0.000
Aps_3478	0.463	0.507	0.630	0.422	0.493	0.489	0.277	0.404	0.340	0.341	0.455	0.455	0.579	0.494	0.632	0.431	0.497	0.583	0.720	0.409	0.463	0.581	0.492	0.419
Aps_3561	0.315	0.440	0.481	0.333	0.449	0.533	0.167	0.281	0.289	0.295	0.421	0.455	0.075	0.141	0.150	0.042	0.081	0.083	0.000	0.000	0.000	0.000	0.000	0.000
Aps_3879	0.396	0.488	0.542	0.444	0.499	0.444	0.277	0.404	0.383	0.261	0.391	0.386	0.313	0.435	0.425	0.278	0.407	0.444	0.375	0.475	0.550	0.360	0.466	0.442
Aps_3936	0.370	0.475	0.519	0.536	0.503	0.452	0.713	0.414	0.277*	0.640	0.466	0.488	0.400	0.486	0.600	0.453	0.503	0.406	0.355	0.464	0.553	0.419	0.492	0.512

Aps_4254	0.056	0.107	0.111	0.022	0.044	0.044	0.011	0.021	0.021	0.034	0.067	0.068	0.175	0.292	0.250	0.083	0.155	0.167	0.061	0.116	0.122	0.012	0.023	0.023
Aps_4334	0.087	0.162	0.174	0.200	0.324	0.356	0.106	0.192	0.213	0.158	0.269	0.211	0.115	0.207	0.231	0.097	0.178	0.139	0.024	0.048	0.049	0.000	0.000	0.000
Aps_4413	0.442	0.503	0.577	0.444	0.499	0.533	0.723	0.404	0.468	0.795	0.329	0.318	0.300	0.425	0.450	0.236	0.366	0.361	0.012	0.024	0.024	0.070	0.131	0.140
Aps_4437	0.519	0.509	0.519	0.415	0.491	0.488	0.384	0.479	0.442	0.407	0.488	0.535	0.300	0.425	0.450	0.314	0.437	0.457	0.365	0.470	0.405	0.302	0.427	0.465
Aps_4891	0.192	0.317	0.385	0.222	0.350	0.356	0.394	0.482	0.489	0.352	0.462	0.477	0.063	0.119	0.125	0.111	0.200	0.222	0.000	0.000	0.000	0.000	0.000	0.000
Aps_5039	0.278	0.409	0.333	0.233	0.362	0.422	0.479	0.504	0.489	0.250	0.380	0.357	0.125	0.222	0.200	0.056	0.106	0.111	0.013	0.025	0.025	0.000	0.000	0.000
Aps_5087	0.065	0.125	0.043	0.011	0.022	0.022	0.021	0.042	0.043	0.057	0.108	0.114	0.051	0.099	0.103	0.083	0.155	0.111	0.000	0.000	0.000	0.035	0.068	0.070
Aps_5166	0.000	0.000	0.000	0.000	0.000	0.000	0.021	0.042	0.043	0.034	0.067	0.068	0.000	0.000	0.000	0.014	0.028	0.028	0.000	0.000	0.000	0.000	0.000	0.000
Aps_5177	0.115	0.208	0.231	0.211	0.337	0.378	0.149	0.256	0.255	0.193	0.315	0.295	0.231	0.360	0.462	0.243	0.373	0.429	0.305	0.429	0.463	0.233	0.361	0.372
Aps_5440	0.167	0.283	0.333	0.200	0.324	0.356	0.053	0.102	0.106	0.068	0.129	0.136	0.100	0.182	0.150	0.111	0.200	0.222	0.220	0.347	0.341	0.128	0.226	0.256
Aps_5722	0.409	0.495	0.545	0.100	0.182	0.200	0.085	0.157	0.170	0.140	0.243	0.186	0.487	0.506	0.658	0.271	0.401	0.371	0.207	0.333	0.366	0.256	0.385	0.465
Aps_5756	0.148	0.257	0.296	0.056	0.106	0.111	0.170	0.286	0.255	0.205	0.329	0.364	0.388	0.481	0.475	0.306	0.430	0.389	0.012	0.024	0.024	0.023	0.046	0.047
Aps_5844	0.111	0.201	0.222	0.179	0.297	0.262	0.106	0.192	0.043*	0.159	0.271	0.091*	0.138	0.240	0.225	0.139	0.243	0.111*	0.110	0.198	0.122	0.128	0.226	0.116*
Aps_5849	0.111	0.201	0.148	0.193	0.315	0.341	0.085	0.157	0.128	0.193	0.315	0.295	0.179	0.298	0.359	0.143	0.248	0.171	0.110	0.198	0.220	0.128	0.226	0.209
Aps_6200	0.315	0.440	0.481	0.182	0.301	0.318	0.340	0.454	0.340	0.477	0.505	0.591	0.288	0.415	0.475	0.441	0.500	0.529	0.159	0.270	0.268	0.151	0.260	0.256
Aps_6269	0.327	0.449	0.500	0.278	0.406	0.422	0.447	0.500	0.553	0.386	0.480	0.545	0.475	0.505	0.400	0.472	0.505	0.444	0.427	0.495	0.561	0.360	0.466	0.488
Aps_6735	0.074	0.140	0.074	0.033	0.065	0.022*	0.011	0.021	0.021	0.023	0.045	0.045	0.000	0.000	0.000	0.000	0.000	0.000	0.073	0.137	0.146	0.035	0.068	0.070
Aps_7032	0.019	0.037	0.037	0.044	0.086	0.089	0.043	0.082	0.085	0.080	0.148	0.114	0.025	0.049	0.050	0.042	0.081	0.083	0.024	0.048	0.049	0.081	0.151	0.116
Aps_7142	0.481	0.509	0.519	0.422	0.493	0.489	0.255	0.384	0.511	0.295	0.421	0.500	0.351	0.462	0.432	0.417	0.493	0.333*	0.338	0.453	0.475	0.381	0.477	0.381
Aps_7176	0.259	0.391	0.370	0.167	0.281	0.289	0.096	0.175	0.149	0.136	0.238	0.227	0.189	0.311	0.270	0.208	0.335	0.306	0.244	0.373	0.390	0.321	0.441	0.405
Aps_7795	0.500	0.511	0.417	0.589	0.490	0.467	0.223	0.351	0.404	0.307	0.430	0.523	0.188	0.309	0.375	0.194	0.318	0.278	0.183	0.303	0.317	0.244	0.373	0.395
Aps_8402	0.093	0.171	0.111	0.144	0.250	0.200	0.096	0.175	0.191	0.159	0.271	0.273	0.138	0.240	0.175	0.069	0.131	0.139	0.275	0.404	0.550	0.321	0.441	0.500
Aps_8845	0.111	0.201	0.222	0.389	0.481	0.422	0.106	0.192	0.085	0.058	0.111	0.070	0.000	0.000	0.000	0.028	0.055	0.056	0.012	0.024	0.024	0.000	0.000	0.000
Aps_8848	0.037	0.073	0.074	0.011	0.022	0.022	0.000	0.000	0.000	0.000	0.000	0.000	0.100	0.182	0.150	0.086	0.159	0.171	0.049	0.094	0.098	0.035	0.068	0.070
Aps_8879	0.096	0.177	0.115	0.022	0.044	0.044	0.089	0.164	0.133	0.179	0.297	0.119*	0.118	0.212	0.184	0.078	0.146	0.156	0.013	0.025	0.025	0.012	0.023	0.023
Aps_8962	0.593	0.492	0.444	0.678	0.442	0.422	0.442	0.499	0.465	0.455	0.502	0.455	0.588	0.491	0.375	0.441	0.500	0.294*	0.705	0.421	0.385	0.762	0.367	0.333
Aps_9107	0.259	0.391	0.444	0.239	0.368	0.250*	0.021	0.042	0.043	0.093	0.171	0.140	0.163	0.276	0.275	0.222	0.351	0.333	0.100	0.182	0.200	0.058	0.111	0.116
Aps_9232	0.135	0.238	0.192	0.278	0.406	0.422	0.053	0.102	0.106	0.057	0.108	0.114	0.063	0.119	0.125	0.111	0.200	0.167	0.073	0.137	0.146	0.093	0.171	0.186
Aps_9283	0.093	0.171	0.185	0.078	0.145	0.156	0.021	0.042	0.043	0.045	0.088	0.091	0.188	0.309	0.325	0.208	0.335	0.250	0.073	0.137	0.146	0.047	0.090	0.093
Aps_9493	0.056	0.107	0.111	0.033	0.065	0.067	0.149	0.256	0.255	0.091	0.167	0.182	0.115	0.207	0.179	0.171	0.288	0.286	0.073	0.137	0.000*	0.095	0.174	0.143
Aps_9518	0.093	0.171	0.185	0.078	0.145	0.111	0.000	0.000	0.000	0.095	0.174	0.190	0.038	0.073	0.075	0.042	0.081	0.083	0.154	0.264	0.256	0.179	0.297	0.262
Aps_9753	0.241	0.372	0.333	0.433	0.497	0.511	0.213	0.339	0.298	0.193	0.315	0.250	0.113	0.202	0.175	0.111	0.200	0.222	0.013	0.025	0.025	0.023	0.046	0.047
Aps_9757	0.241	0.372	0.333	0.372	0.473	0.538	0.380	0.477	0.457	0.372	0.473	0.465	0.264	0.394	0.528	0.403	0.488	0.528	0.110	0.198	0.171	0.151	0.260	0.256
Aps_9847	0.019	0.037	0.037	0.170	0.286	0.295	0.096	0.175	0.149	0.136	0.238	0.227	0.013	0.025	0.025	0.181	0.300	0.250	0.566	0.498	0.711	0.512	0.506	0.488
Aps_9958	0.037	0.073	0.074	0.100	0.182	0.200	0.085	0.157	0.170	0.227	0.355	0.409	0.100	0.182	0.150	0.143	0.248	0.229	0.024	0.048	0.000*	0.081	0.151	0.116
Aps_9979	0.593	0.492	0.593	0.278	0.406	0.378	0.149	0.256	0.255	0.190	0.312	0.333	0.308	0.432	0.462	0.472	0.505	0.500	0.500	0.506	0.538	0.593	0.488	0.535
Aps_9982	0.100	0.184	0.200	0.022	0.044	0.044	0.043	0.082	0.085	0.034	0.067	0.068	0.013	0.025	0.025	0.042	0.081	0.083	0.061	0.116	0.122	0.093	0.171	0.186
Aps_10095	0.852	0.257	0.148	0.867	0.234	0.222	0.968	0.062	0.064	0.881	0.212	0.238	0.885	0.207	0.231	0.486	0.507	0.528	0.976	0.048	0.049	0.988	0.023	0.023
Aps_10259	0.037	0.073	0.074	0.000	0.000	0.000	0.000	0.000	0.000	0.000	0.000	0.000	0.025	0.049	0.000	0.014	0.028	0.028	0.000	0.000	0.000	0.000	0.000	0.000
Aps_10291	0.019	0.037	0.037	0.022	0.044	0.044	0.170	0.286	0.298	0.159	0.271	0.273	0.113	0.202	0.125	0.028	0.055	0.056	0.012	0.024	0.024	-	-	-
Aps_10338	0.111	0.201	0.148	0.333	0.449	0.356	0.085	0.157	0.128	0.080	0.148	0.159	0.038	0.073	0.075	0.125	0.222	0.250	0.012	0.024	0.024	0.000	0.000	0.000

Aps_10554	0.556	0.503	0.519	0.522	0.505	0.556	0.426	0.494	0.383	0.419	0.492	0.512	0.425	0.495	0.500	0.286	0.414	0.343	0.295	0.421	0.436	0.221	0.348	0.395
Aps_11191	0.222	0.352	0.370	0.222	0.350	0.311	0.170	0.286	0.298	0.148	0.255	0.295	0.263	0.392	0.425	0.222	0.351	0.278	0.244	0.373	0.390	0.256	0.385	0.419
Aps_11218	0.111	0.201	0.222	0.034	0.067	0.068	0.067	0.126	0.133	0.075	0.141	0.150	0.063	0.119	0.125	0.015	0.029	0.029	0.113	0.202	0.225	0.116	0.208	0.186
Aps_11832	0.148	0.257	0.222	0.167	0.281	0.244	0.383	0.478	0.468	0.302	0.427	0.372	0.218	0.345	0.333	0.153	0.263	0.306	0.050	0.096	0.100	0.023	0.046	0.047
Aps_12281	0.093	0.171	0.185	0.067	0.126	0.133	0.011	0.021	0.021	0.011	0.023	0.023	0.013	0.025	0.025	0.000	0.000	0.000	0.000	0.000	0.000	0.000	0.000	0.000
Aps_12422	0.241	0.372	0.333	0.318	0.439	0.500	0.223	0.351	0.362	0.307	0.430	0.432	0.449	0.501	0.487	0.486	0.507	0.528	0.395	0.484	0.474	0.430	0.496	0.628
Aps_14730	0.000	0.000	0.000	0.048	0.092	0.048	0.011	0.021	0.021	0.000	0.000	0.000	0.121	0.216	0.060*	0.118	0.211	0.117*	0.000	0.000	0.000	0.000	0.000	0.000
Aps_14949	0.540	0.507	0.600	0.344	0.457	0.422	0.160	0.271	0.234	0.170	0.286	0.341	0.313	0.435	0.275*	0.389	0.482	0.444	0.329	0.447	0.463	0.256	0.385	0.326
Aps_15051	0.111	0.201	0.222	0.078	0.145	0.156	0.011	0.021	0.021	0.011	0.023	0.023	0.118	0.212	0.237	0.153	0.263	0.306	0.037	0.071	0.073	0.060	0.113	0.119
Aps_15194	0.426	0.498	0.481	0.289	0.415	0.400	0.489	0.505	0.553	0.432	0.496	0.455	0.588	0.491	0.675	0.444	0.501	0.500	0.463	0.503	0.585	0.465	0.503	0.512
Aps_15910	0.333	0.453	0.444	0.267	0.396	0.400	0.255	0.384	0.383	0.364	0.468	0.500	0.463	0.503	0.475	0.417	0.493	0.333*	0.163	0.276	0.275	0.256	0.385	0.465
Aps_16114	0.407	0.492	0.519	0.411	0.490	0.467	0.362	0.467	0.468	0.360	0.466	0.442	0.638	0.468	0.525	0.400	0.487	0.571	0.195	0.318	0.341	0.221	0.348	0.349
Aps_16139	0.000	0.000	0.000	0.011	0.022	0.022	0.064	0.121	0.128	0.024	0.048	0.049	0.075	0.141	0.150	0.074	0.138	0.147	0.095	0.174	0.189	0.105	0.190	0.163
Aps_16862	0.000	0.000	0.000	0.000	0.000	0.000	0.000	0.000	0.000	0.000	0.000	0.000	0.013	0.025	0.025	0.111	0.200	0.222	0.488	0.506	0.634	0.395	0.484	0.419
Aps_19806	0.115	0.208	0.154	0.244	0.374	0.444	0.128	0.225	0.255	0.091	0.167	0.136	0.038	0.073	0.075	0.097	0.178	0.194	0.050	0.096	0.100	0.140	0.243	0.233
Aps_21003	0.056	0.107	0.111	0.044	0.086	0.089	0.202	0.326	0.319	0.239	0.368	0.432	0.050	0.096	0.100	0.069	0.131	0.139	0.061	0.116	0.122	0.058	0.111	0.116
Aps_22011	0.423	0.498	0.462	0.622	0.475	0.489	0.702	0.423	0.468	0.545	0.502	0.455	0.450	0.501	0.450	0.319	0.441	0.361	0.732	0.397*	0.244	0.744	0.385	0.372
Aps_22309	0.130	0.230	0.259	0.216	0.342	0.295	0.468	0.503	0.383	0.500	0.506	0.455	0.275	0.404	0.450	0.278	0.407	0.389	0.183	0.303	0.268	0.128	0.226	0.256
Aps_25940	0.074	0.140	0.148	0.067	0.126	0.133	0.085	0.157	0.170	0.045	0.088	0.091	0.013	0.025	0.025	0.059	0.112	0.118	0.000	0.000	0.000	0.000	0.000	0.000
