# Supplementary material for: Discovery and characterization of single nucleotide polymorphisms in two anadromous alosine fishes of conservation concern
Source: Ecol Evol. 2017 Jul 18;7(17):6638–48. doi: 10.1002/ece3.3215 (PMC5587496; doi:10.1002/ece3.3215)
Supplement: Supplementary file 6 [file ECE3-7-6638-s006.pdf]

Table S5. Blueback allele frequencies, expected, and observed heterozygosities by locus. Allele frequencies are standardized to the minor allele in the Monument River. *** = out of Hardy Weinberg equlibrium**

	Margaree N = 40			Peticodiac N = 27			East Machias N = 45			Kennebec N = 47			Mystic N = 44			Monument N = 47			Delaware N = 47		
Assay name	Allele Freq.	He	Ho	Allele Freq.	He	Ho	Allele Freq.	He	Ho	Allele Freq.	He	Ho	Allele Freq.	He	Ho	Allele Freq.	He	Ho	Allele Freq.	He	Ho
Aae_2	0.213	0.339	0.375	0.463	0.507	0.481	0.333	0.449	0.489	0.315	0.436	0.500	0.341	0.455	0.591	0.309	0.431	0.447	0.447	0.500	0.468
Aae_71	0.663	0.453	0.275*	0.667	0.454	0.500	0.756	0.374	0.267	0.598	0.486	0.587	0.261	0.391	0.341	0.440	0.499	0.452	-	-	-
Aae_136	0.100	0.182	0.150	0.074	0.140	0.148	0.089	0.164	0.133	0.109	0.196	0.217	0.034	0.067	0.068	0.011	0.021	0.021	0.043	0.082	0.085
Aae_219	0.325	0.444	0.450	0.074	0.140	0.148	0.233	0.362	0.378	0.149	0.256	0.298	0.034	0.067	0.068	0.074	0.139	0.149	0.191	0.313	0.383
Aae_258	0.038	0.073	0.075	0.056	0.107	0.111	0.033	0.065	0.067	0.021	0.042	0.043	0.151	0.260	0.256	0.250	0.379	0.413	0.064	0.121	0.085
Aae_304	0.425	0.495	0.450	0.204	0.331	0.333	0.156	0.266	0.311	0.170	0.286	0.340	0.227	0.355	0.409	0.309	0.431	0.489	0.426	0.494	0.340*
Aae_430	0.400	0.486	0.450	0.152	0.264	0.304	0.261	0.391	0.341	0.380	0.477	0.630	0.455	0.502	0.636	0.426	0.494	0.511	0.149	0.256	0.255
Aae_459	0.150	0.258	0.300	0.333	0.453	0.593	0.356	0.463	0.356	0.245	0.374	0.362	0.398	0.485	0.477	0.372	0.472	0.447	0.309	0.431	0.447
Aae_462	0.025	0.049	0.050	0.000	0.000	0.000	0.044	0.086	0.089	0.032	0.062	0.064	0.148	0.255	0.250	0.256	0.385	0.422	0.043	0.082	0.043
Aae_554	0.100	0.182	0.150	0.000	0.000	0.000	0.044	0.086	0.089	0.117	0.209	0.234	0.310	0.433	0.429	0.096	0.175	0.191	0.277	0.404	0.298
Aae_694	0.125	0.222	0.250	0.056	0.107	0.111	0.078	0.145	0.156	0.245	0.374	0.362	0.068	0.129	0.136	0.000	0.000	0.000	0.213	0.339	0.298
Aae_928	0.100	0.182	0.200	0.148	0.257	0.296	0.100	0.182	0.200	0.138	0.241	0.234	0.182	0.301	0.318	0.319	0.439	0.426	0.098	0.178	0.196
Aae_1107	0.100	0.182	0.200	0.093	0.171	0.185	0.122	0.217	0.244	0.085	0.157	0.128	0.250	0.379	0.227*	0.170	0.286	0.340	0.223	0.351	0.362
Aae_1144	0.063	0.119	0.125	0.111	0.201	0.222	0.289	0.415	0.444	0.222	0.350	0.311	0.102	0.186	0.205	0.000	0.000	0.000	0.160	0.271	0.319
Aae_1275	0.438	0.498	0.325*	0.444	0.503	0.593	0.364	0.468	0.409	0.217	0.344	0.304	0.250	0.379	0.318	0.309	0.431	0.404	0.234	0.362	0.340
Aae_1287	0.225	0.353	0.450	0.185	0.307	0.370	0.311	0.433	0.444	0.309	0.431	0.362	0.125	0.221	0.250	0.138	0.241	0.234	0.255	0.384	0.468
Aae_1354	0.350	0.461	0.300*	0.231	0.362	0.385	0.400	0.485	0.444	0.404	0.487	0.468	0.341	0.455	0.500	0.457	0.502	0.532	0.372	0.472	0.532
Aae_1454	-	-	-	-	-	-	0.261	0.391	0.432	0.326	0.444	0.652	0.352	0.462	0.659	0.043	0.082	0.085	0.446	0.500	0.717
Aae_1482	0.038	0.073	0.075	0.111	0.201	0.222	0.067	0.126	0.133	0.043	0.082	0.085	0.081	0.151	0.163	0.167	0.281	0.333	0.053	0.102	0.106
Aae_1504	0.325	0.444	0.500	0.056	0.107	0.111	0.322	0.442	0.511	0.463	0.503	0.488	0.227	0.355	0.455	0.170	0.286	0.255	0.489	0.505	0.340
Aae_1605	0.300	0.425	0.400	0.269	0.401	0.385	0.278	0.406	0.467	0.383	0.478	0.511	0.102	0.186	0.205	0.117	0.209	0.191	0.021	0.042	0.043
Aae_1792	0.088	0.162	0.125	0.000	0.000	0.000	0.078	0.145	0.111	0.128	0.225	0.213	0.114	0.204	0.227	0.053	0.102	0.106	0.266	0.395	0.404
Aae_2120	0.038	0.073	0.025*	0.000	0.000	0.000	0.011	0.022	0.022	0.111	0.200	0.133	0.000	0.000	0.000	0.000	0.000	0.000	0.111	0.200	0.133
Aae_2136	0.013	0.025	0.025	0.000	0.000	0.000	0.000	0.000	0.000	0.021	0.042	0.043	0.000	0.000	0.000	0.032	0.062	0.064	0.098	0.178	0.152
Aae_2146	0.300	0.425	0.400	0.241	0.372	0.407	0.256	0.385	0.422	0.138	0.241	0.149*	0.114	0.204	0.227	0.170	0.286	0.255	0.255	0.384	0.340
Aae_2367	0.590	0.490	0.462	0.204	0.331	0.407	0.556	0.499	0.444	0.521	0.504	0.574	0.477	0.505	0.455	0.432	0.496	0.455	0.415	0.491	0.574
Aae_2394	-	-	-	-	-	-	0.233	0.361	0.419	0.207	0.331	0.370	0.345	0.458	0.500	0.175	0.292	0.200	0.096	0.175	0.191
Aae_2420	0.225	0.353	0.400	0.426	0.498	0.556	0.318	0.439	0.409	0.330	0.447	0.447	0.012	0.024	0.024	0.054	0.104	0.109	0.074	0.139	0.149
Aae_2483	0.063	0.119	0.125	0.037	0.073	0.074	0.156	0.266	0.311	0.141	0.245	0.283	0.291	0.417	0.488	0.213	0.339	0.340	0.326	0.444	0.465
Aae_2650	0.115	0.207	0.231	0.000	0.000	0.000	0.178	0.296	0.222	0.202	0.326	0.319	0.037	0.071	0.073	0.130	0.229	0.261	0.011	0.021	0.021
Aae_2668	0.600	0.486	0.400	0.500	0.509	0.630	0.667	0.449	0.489	0.641	0.465	0.413	0.318	0.439	0.318	0.394	0.482	0.489	0.383	0.478	0.426
Aae_2726	0.088	0.162	0.125	0.148	0.257	0.222	0.089	0.164	0.133	0.106	0.192	0.170	0.080	0.148	0.068*	0.076	0.142	0.152	0.074	0.139	0.149
Aae_2937	0.325	0.444	0.400	0.259	0.391	0.444	0.411	0.490	0.511	0.380	0.477	0.500	0.232	0.360	0.415	0.223	0.351	0.404	0.426	0.494	0.553
Aae_2984	0.338	0.453	0.575	0.308	0.434	0.385	0.318	0.439	0.318	0.326	0.444	0.348	0.607	0.483	0.548	0.457	0.502	0.565	0.500	0.505	0.532

Aae_3391	0.013	0.025	0.025	0.000	0.000	0.000	0.078	0.145	0.156	0.053	0.102	0.106	0.068	0.129	0.136	0.011	0.021	0.021	0.074	0.139	0.149
Aae_3432	0.100	0.182	0.200	0.019	0.037	0.037	0.256	0.385	0.378	0.174	0.290	0.304	0.045	0.088	0.091	0.000	0.000	0.000	0.096	0.175	0.149
Aae_3449	0.000	0.000	0.000	0.192	0.317	0.308	0.067	0.126	0.133	0.053	0.102	0.106	0.205	0.329	0.318	0.174	0.290	0.304	0.053	0.102	0.106
Aae_3704	0.775	0.353	0.400	0.556	0.503	0.667	0.822	0.296	0.311	0.815	0.305	0.326	0.489	0.505	0.477	0.457	0.502	0.532	0.723	0.404	0.426
Aae_3706	0.600	0.486	0.550	0.444	0.503	0.370	0.602	0.485	0.432	0.543	0.502	0.617	0.386	0.480	0.455	0.447	0.500	0.511	0.255	0.384	0.340
Aae_3749	0.050	0.096	0.100	0.000	0.000	0.000	0.011	0.022	0.022	0.021	0.042	0.043	0.080	0.148	0.159	0.043	0.082	0.085	0.011	0.021	0.021
Aae_3768	0.375	0.475	0.300*	0.479	0.510	0.542	0.411	0.490	0.467	0.500	0.505	0.489	0.557	0.499	0.477	0.447	0.500	0.426	0.433	0.497	0.467
Aae_3882	0.350	0.461	0.400	0.204	0.331	0.407	0.400	0.485	0.578	0.404	0.487	0.511	0.443	0.499	0.523	0.436	0.497	0.489	0.330	0.447	0.574
Aae_3942	0.113	0.202	0.075	0.093*	0.171	0.185	0.111	0.200	0.222	0.076	0.142	0.152	0.093	0.171	0.186	0.138	0.241	0.234	0.021	0.042	0.043
Aae_4025	0.075	0.141	0.150	0.000	0.000	0.000	0.022	0.044	0.044	0.011	0.021	0.021	0.557	0.499	0.886	0.500	0.505	0.915	0.074	0.139	0.149
Aae_4063	0.313	0.435	0.375	0.185	0.307	0.296	0.178	0.296	0.267	0.266	0.395	0.404	0.417	0.492	0.548	0.394	0.482	0.447	0.620	0.477	0.500
Aae_4154	0.225	0.353	0.350	0.370	0.475	0.370	0.278	0.406	0.467	0.383	0.478	0.468	0.081	0.151	0.163	0.191	0.313	0.383	0.245	0.374	0.404
Aae_4191	0.175	0.292	0.250	0.241	0.372	0.407	0.089	0.164	0.133	0.096	0.175	0.191	0.125	0.221	0.205	0.128	0.225	0.255	0.128	0.225	0.255
Aae_4223	0.100	0.182	0.150	0.111	0.201	0.222	-	-	-	0.043	0.082	0.085	0.045	0.088	0.091	0.213	0.339	0.298	0.000	0.000	0.000
Aae_4247	0.338	0.453	0.475	0.462	0.507	0.462	0.411	0.490	0.511	0.245	0.374	0.319	0.417	0.492	0.548	0.457	0.502	0.532	0.389	0.481	0.556
Aae_4287	0.400	0.486	0.500	0.231	0.362	0.462	0.311	0.433	0.356	0.351	0.461	0.489	0.386	0.480	0.455	0.319	0.439	0.553	0.330	0.447	0.489
Aae_4366	0.000	0.000	0.000	0.056	0.107	0.111	0.000	0.000	0.000	0.000	0.000	0.000	0.000	0.000	0.000	0.000	0.000	0.000	0.000	0.000	0.000
Aae_4389	0.288	0.415	0.425	0.630	0.475	0.296	0.267	0.396	0.444	0.340	0.454	0.383	0.136	0.238	0.182	0.436	0.497	0.149	0.106	0.192	0.170
Aae_4472	0.250	0.380	0.300	0.167	0.283	0.333	0.244	0.374	0.400	0.191	0.313	0.298	0.250	0.379	0.364	0.223	0.351	0.447	0.117	0.209	0.234
Aae_4602	0.275	0.404	0.300	0.074	0.140	0.148	0.244	0.374	0.400	0.277	0.404	0.383	0.023	0.045	0.045	0.096	0.175	0.191	0.394	0.482	0.532
Aae_4733	0.063	0.119	0.125	0.000	0.000	0.000	0.044	0.086	0.089	0.043	0.082	0.043	0.034	0.067	0.068	0.000	0.000	0.000	0.096	0.175	0.191
Aae_4940	0.238	0.367	0.275	0.167	0.283	0.259	0.333	0.449	0.489	0.245	0.374	0.319	0.128	0.226	0.256	0.064	0.121	0.128	0.223	0.351	0.404
Aae_4985	0.200	0.324	0.200*	0.160	0.274	0.320	0.267	0.396	0.400	0.359	0.465	0.587	0.193	0.315	0.386	0.032	0.062	0.021*	0.383	0.478	0.511
Aae_5113	0.188	0.309	0.375	0.500	0.509	0.630	0.156	0.266	0.311	0.185	0.305	0.326	0.155	0.265	0.262	0.245	0.374	0.362	0.053	0.102	0.106
Aae_5177	0.063	0.119	0.125	0.093	0.171	0.185	0.067	0.126	0.133	0.065	0.123	0.130	0.080	0.148	0.159	0.053	0.102	0.064	0.085	0.157	0.170
Aae_5262	0.050	0.096	0.100	0.019	0.037	0.037	0.022	0.044	0.044	0.085	0.157	0.170	0.080	0.148	0.114	0.011	0.021	0.021	0.053	0.102	0.106
Aae_5492	0.163	0.276	0.225	0.148	0.257	0.296	0.156	0.266	0.222	0.181	0.299	0.362	0.273	0.401	0.364	0.160	0.271	0.319	0.085	0.157	0.128
Aae_5563	0.250	0.380	0.200*	0.200	0.327	0.240	0.227	0.355	0.273	0.141	0.245	0.196	0.186	0.306	0.279	0.128	0.225	0.255	0.138	0.241	0.149*
Aae_5703	0.238	0.367	0.375	0.019	0.037	0.037	0.222	0.350	0.311	0.117	0.209	0.191	0.102	0.186	0.205	0.043	0.082	0.085	0.287	0.414	0.404
Aae_5737	0.205	0.330	0.308	0.180	0.301	0.280	-	-	-	0.120	0.213	0.239	0.114	0.204	0.182	0.117	0.209	0.234	0.170	0.286	0.255
Aae_5780	0.225	0.353	0.200*	0.426	0.498	0.481	0.400	0.485	0.489	0.272	0.400	0.500	0.068	0.129	0.136	0.096	0.175	0.106*	0.256	0.385	0.378
Aae_5826	0.413	0.491	0.575	0.288	0.419	0.577	0.411	0.490	0.511	0.383	0.478	0.468	0.233	0.361	0.279	0.479	0.504	0.489	0.160	0.271	0.319
Aae_5833	0.113	0.202	0.175	0.096	0.177	0.192	0.100	0.182	0.200	0.074	0.139	0.106	0.136	0.238	0.182	0.021	0.042	0.000	0.223	0.351	0.362
Aae_5869	0.588	0.491	0.425	0.407	0.492	0.815	0.444	0.499	0.356*	0.489	0.505	0.596	0.443	0.499	0.523	0.415	0.491	0.362	0.511	0.505	0.468
Aae_5886	0.051	0.099	0.103	0.000	0.000	0.000	0.056	0.106	0.111	0.043	0.082	0.085	0.034	0.067	0.068	0.021	0.042	0.000	0.000	0.000	0.000
Aae_5919	0.238	0.367	0.425	0.300	0.429	0.600	0.322	0.442	0.378	0.426	0.494	0.596	0.432	0.496	0.545	0.351	0.461	0.532	0.319	0.439	0.340
Aae_6021	0.641	0.466	0.513	0.688	0.439	0.542	0.690	0.433	0.476	0.638	0.467	0.553	0.698	0.427	0.326	0.436	0.497	0.489	0.681	0.439	0.553
Aae_6083	0.150	0.258	0.300	0.241	0.372	0.481	0.159	0.271	0.227	0.130	0.229	0.261	0.305	0.429	0.317	0.415	0.491	0.574	0.255	0.384	0.383
Aae_6273	0.400	0.486	0.450	0.712	0.419	0.577	0.330	0.447	0.477	0.337	0.452	0.500	0.214	0.341	0.333	0.120	0.213	0.196	0.117	0.209	0.191
Aae_6302	0.350	0.461	0.450	0.148	0.257	0.222	0.244	0.374	0.311	0.255	0.384	0.383	0.119	0.212	0.238	0.065	0.123	0.087	0.283	0.410	0.304
Aae_6327	0.263	0.392	0.375	0.370	0.475	0.593	0.456	0.502	0.511	0.511	0.505	0.468	0.273	0.401	0.409	0.500	0.505	0.404	0.160	0.271	0.277

Aae_6571	0.688	0.435	0.475	0.523	0.511	0.773	0.722	0.406	0.333	0.723	0.404	0.340	0.625	0.474	0.432	0.426	0.494	0.426	0.717	0.410	0.391
Aae_6703	0.425	0.495	0.400	0.565	0.502	0.696	0.444	0.499	0.489	0.532	0.503	0.468	0.500	0.506	0.409	0.457	0.502	0.489	0.543	0.502	0.574
Aae_6727	0.038	0.073	0.075	0.093	0.171	0.185	0.011	0.022	0.022	0.032	0.062	0.064	0.000	0.000	0.000	0.000	0.000	0.000	0.106	0.192	0.170
Aae_6831	0.138	0.240	0.275	0.130	0.230	0.259	0.111	0.200	0.133	0.106	0.192	0.213	0.034	0.067	0.068	0.160	0.271	0.234	0.053	0.102	0.106
Aae_7011	0.038	0.073	0.075	0.000	0.000	0.000	0.011	0.022	0.022	0.011	0.021	0.021	0.045	0.088	0.091	0.000	0.000	0.000	0.138	0.241	0.234
Aae_7040	0.375	0.475	0.500	0.154	0.265	0.231	0.267	0.396	0.356	0.223	0.351	0.319	0.256	0.385	0.465	0.287	0.414	0.532	0.138	0.241	0.234
Aae_7090	0.000	0.000	0.000	0.000	0.000	0.000	0.044	0.086	0.089	0.011	0.021	0.021	0.023	0.045	0.045	0.064	0.121	0.128	0.054	0.104	0.065
Aae_7200	0.238	0.367	0.375	0.259	0.391	0.444	0.122	0.217	0.244	0.239	0.368	0.261	0.455	0.502	0.455	0.351	0.461	0.489	0.170	0.286	0.298
Aae_7269	0.225	0.353	0.350	0.352	0.465	0.481	0.422	0.493	0.444	0.500	0.505	0.532	0.466	0.503	0.568	0.489	0.505	0.553	0.245	0.374	0.319
Aae_7796	0.150	0.258	0.250	0.111	0.201	0.222	0.111	0.200	0.222	0.149	0.256	0.255	0.057	0.108	0.114	0.043	0.084	0.087	0.106	0.192	0.213
Aae_8252	-	-	-	-	-	-	0.111	0.200	0.178	0.096	0.175	0.191	0.279	0.407	0.279*	0.185	0.305	0.370	0.181	0.299	0.319
Aae_8427	0.256	0.386	0.256*	0.037	0.073	0.074	0.256	0.385	0.289	0.402	0.486	0.413	0.136	0.238	0.182	0.250	0.379	0.326	0.426	0.494	0.383
Aae_8435	0.450	0.501	0.450	0.460	0.507	0.600	0.522	0.505	0.511	0.649	0.461	0.362	0.284	0.411	0.477	0.444	0.499	0.489	0.585	0.491	0.447
Aae_8734	0.075	0.141	0.150	0.056	0.107	0.111	0.022	0.044	0.044	0.065	0.123	0.130	0.080	0.148	0.159	0.096	0.175	0.191	0.160	0.271	0.319
Aae_9052	0.013	0.025	0.025	0.130	0.230	0.185	0.067	0.126	0.133	0.021	0.042	0.043	0.011	0.023	0.023	0.000	0.000	0.000	0.032	0.062	0.064
Aae_9057	0.250	0.380	0.300	0.565	0.502	0.522	0.200	0.324	0.400	0.272	0.400	0.370	0.193	0.315	0.250	0.148	0.255	0.295	0.289	0.415	0.400
Aae_10384	0.375	0.475	0.350	0.500	0.509	0.704	0.289	0.415	0.489	0.315	0.436	0.500	0.233	0.361	0.372	0.266	0.395	0.404	0.426	0.494	0.426
Aae_10678	0.088	0.162	0.175	0.058	0.111	0.115	0.178	0.296	0.356	0.149	0.256	0.255	0.093	0.171	0.186	0.021	0.042	0.043	0.021	0.042	0.043
Aae_10801	0.163	0.276	0.325	0.056	0.107	0.111	0.067	0.126	0.133	0.074	0.139	0.149	0.045	0.088	0.091	0.223	0.351	0.447	0.170	0.286	0.340
Aae_11731	0.125	0.222	0.200	0.019	0.037	0.037	0.222	0.350	0.356	0.191	0.313	0.298	0.274	0.402	0.452	0.289	0.415	0.400	0.217	0.344	0.261
Aae_11754	0.063	0.119	0.125	0.077	0.145	0.154	0.102	0.186	0.205	0.149	0.256	0.170*	0.409	0.489	0.500	0.277	0.404	0.468	0.064	0.121	0.128

Table S5. Blueback allele frequencies, expected, and observed heterozygosities by locus. Allele frequencies are standardized to the minor allele in the Monument River.
*** = out of Hardy Weinberg equlibrium**

	Rappahannock N = 42			Savannah N = 47			Altamaha N = 47		
Assay name	Allele Freq.	He	Ho	Allele Freq.	He	Ho	Allele Freq.	He	Ho
Aae_2	0.476	0.505	0.571	0.404	0.487	0.596	0.319	0.439	0.553
Aae_71	0.512	0.506	0.643	0.553	0.500	0.511	0.521	0.504	0.489
Aae_136	0.024	0.047	0.048	0.000	0.000	0.000	0.000	0.000	0.000
Aae_219	0.155	0.265	0.310	0.298	0.423	0.383	0.298	0.423	0.426
Aae_258	0.071	0.134	0.143	0.011	0.021	0.021	0.011	0.021	0.021
Aae_304	0.500	0.506	0.561	0.213	0.339	0.383	0.309	0.431	0.447
Aae_430	0.119	0.212	0.190	0.117	0.209	0.191	0.106	0.192	0.170
Aae_459	0.286	0.413	0.381	0.543	0.502	0.489	0.372	0.472	0.574
Aae_462	0.071	0.134	0.143	0.064	0.121	0.128	0.021	0.042	0.043
Aae_554	0.207	0.333	0.317	0.064	0.121	0.128	0.096	0.175	0.191
Aae_694	0.202	0.327	0.357	0.106	0.192	0.128	0.202	0.326	0.362
Aae_928	0.143	0.248	0.238	0.032	0.062	0.064	0.032	0.062	0.064
Aae_1107	0.274	0.402	0.500	0.160	0.271	0.277	0.234	0.362	0.426
Aae_1144	0.214	0.341	0.381	0.053	0.102	0.106	0.021	0.042	0.043
Aae_1275	0.310	0.433	0.476	0.585	0.491	0.362	0.500	0.505	0.574
Aae_1287	0.321	0.441	0.452	0.106	0.192	0.213	0.053	0.102	0.106
Aae_1354	0.390	0.482	0.488	0.479	0.504	0.532	0.489	0.505	0.511
Aae_1454	0.405	0.488	0.810	0.468	0.503	0.766	0.436	0.497	0.830
Aae_1482	0.095	0.174	0.143	0.033	0.064	0.065	0.053	0.102	0.106
Aae_1504	0.440*	0.499	0.500	0.149	0.256	0.298	0.287	0.414	0.404
Aae_1605	0.071	0.134	0.095	0.074	0.139	0.149	0.043	0.082	0.085
Aae_1792	0.250	0.380	0.450	0.287	0.414	0.362	0.191	0.313	0.340
Aae_2120	0.100	0.182	0.050*	0.044	0.086	0.044	0.065	0.123	0.000
Aae_2136	0.095	0.174	0.190	0.543	0.502	0.532	0.479	0.504	0.532
Aae_2146	0.238	0.367	0.429	0.213	0.339	0.255	0.149	0.256	0.255
Aae_2367	0.429	0.496	0.429	0.196	0.318	0.391	0.217	0.344	0.304
Aae_2394	0.107	0.194	0.214	0.011	0.021	0.021	0.022	0.043	0.043
Aae_2420	0.095	0.174	0.143	0.000	0.000	0.000	0.011	0.021	0.021
Aae_2483	0.310	0.433	0.571	0.213	0.339	0.340	0.255	0.384	0.383
Aae_2650	0.036	0.070	0.071	0.138	0.241	0.277	0.096	0.175	0.191
Aae_2668	0.417	0.492	0.452	0.138	0.241	0.234	0.287	0.414	0.532
Aae_2726	0.071	0.134	0.143	0.011	0.021	0.021	0.053	0.102	0.106
Aae_2937	0.393	0.483	0.405	0.213	0.339	0.298	0.245	0.374	0.362
Aae_2984	0.548	0.501	0.429	0.609	0.482	0.609	0.702	0.423	0.468

Aae_3391	0.036	0.070	0.071	0.021	0.042	0.043	0.011	0.021	0.021
Aae_3432	0.167	0.281	0.333	0.053	0.102	0.106	0.085	0.157	0.170
Aae_3449	0.107	0.194	0.167	0.032	0.062	0.064	0.021	0.042	0.043
Aae_3704	0.798	0.327	0.262	0.424	0.494	0.413	0.479	0.504	0.532
Aae_3706	0.202	0.327	0.310	0.277	0.404	0.298	0.298	0.423	0.468
Aae_3749	0.012	0.024	0.024	0.106	0.192	0.170	0.064	0.121	0.128
Aae_3768	0.415	0.491	0.439	0.564	0.497	0.617	0.564	0.497	0.447
Aae_3882	0.369	0.471	0.643	0.372	0.472	0.319*	0.415	0.491	0.489
Aae_3942	0.060	0.113	0.071	0.085	0.157	0.170	0.032	0.062	0.064
Aae_4025	0.095	0.174	0.190	0.649	0.461	0.447	0.660	0.454	0.511
Aae_4063	0.571	0.496	0.476	0.840	0.271	0.277	0.734	0.395	0.447
Aae_4154	0.354	0.463	0.512	0.766	0.362	0.426	0.745	0.384	0.383
Aae_4191	0.220	0.347	0.341	0.217	0.344	0.348	0.211	0.337	0.333
Aae_4223	0.000	0.000	0.000	0.000	0.000	0.000	0.000	0.000	0.000
Aae_4247	0.274	0.402	0.405	0.713	0.414	0.404	0.702	0.423	0.383
Aae_4287	0.390	0.482	0.439	0.564	0.497	0.532	0.340	0.454	0.468
Aae_4366	0.000	0.000	0.000	0.011	0.021	0.021	0.053	0.102	0.106
Aae_4389	0.095	0.174	0.190	0.117	0.209	0.149	0.170	0.286	0.170*
Aae_4472	0.202	0.327	0.357	0.223	0.351	0.277	0.053	0.102	0.106
Aae_4602	0.345	0.458	0.500	0.404	0.487	0.383	0.436	0.497	0.489
Aae_4733	0.095	0.174	0.190	0.266	0.395	0.277*	0.191	0.313	0.298
Aae_4940	0.179	0.297	0.310	0.202	0.326	0.191*	0.138	0.241	0.234
Aae_4985	0.464	0.503	0.595	0.255	0.384	0.298	0.330	0.447	0.447
Aae_5113	0.048	0.092	0.048	0.128	0.225	0.213	0.021	0.042	0.043
Aae_5177	0.083	0.155	0.071*	0.043	0.082	0.085	0.022	0.043	0.043
Aae_5262	0.036	0.070	0.071	0.128	0.225	0.255	0.191	0.313	0.255
Aae_5492	0.122	0.217	0.195	0.032	0.062	0.064	0.021	0.042	0.043
Aae_5563	0.195	0.318	0.293	0.074	0.139	0.064*	0.149	0.256	0.085*
Aae_5703	0.250	0.380	0.452	0.117	0.209	0.234	0.096	0.175	0.149
Aae_5737	0.119	0.212	0.238	0.277	0.404	0.468	0.309	0.431	0.404
Aae_5780	0.310	0.433	0.429	0.255	0.384	0.468	0.298	0.423	0.383
Aae_5826	0.280	0.409	0.463	0.074	0.139	0.106	0.085	0.157	0.170
Aae_5833	0.274	0.402	0.405	0.106	0.192	0.213	0.053	0.102	0.106
Aae_5869	0.628	0.473	0.538	0.723	0.404	0.426	0.596	0.487	0.596
Aae_5886	0.012	0.024	0.024	0.000	0.000	0.000	0.064	0.121	0.128
Aae_5919	0.317	0.438	0.537	0.340	0.454	0.511	0.500	0.505	0.532
Aae_6021	0.643	0.465	0.429	0.660	0.454	0.426	0.649	0.461	0.574
Aae_6083	0.262	0.391	0.429	0.489	0.505	0.553	0.394	0.482	0.574
Aae_6273	0.131	0.230	0.262	0.149	0.256	0.255	0.223	0.351	0.362
Aae_6302	0.310	0.433	0.381	0.362	0.467	0.426	0.319	0.439	0.426
Aae_6327	0.262	0.391	0.381	0.500	0.505	0.489	0.574	0.494	0.596

Aae_6571	0.726	0.402	0.452	0.617	0.478	0.596	0.777	0.351	0.404
Aae_6703	0.429	0.496	0.476	0.479	0.504	0.574	0.457	0.502	0.489
Aae_6727	0.107	0.194	0.214	0.723	0.404	0.298	0.761	0.368	0.391
Aae_6831	0.071	0.134	0.143	0.000	0.000	0.000	0.000	0.000	0.000
Aae_7011	0.071	0.134	0.095	0.096	0.175	0.149	0.138	0.241	0.277
Aae_7040	0.119	0.212	0.238	0.447	0.500	0.383	0.479	0.504	0.447
Aae_7090	0.012	0.024	0.024	0.021	0.042	0.043	0.032	0.062	0.021*
Aae_7200	0.167	0.281	0.238	0.117	0.209	0.149	0.096	0.175	0.191
Aae_7269	0.179	0.297	0.262	0.035	0.068	0.023	0.067	0.126	0.089
Aae_7796	0.202	0.327	0.357	0.207	0.331	0.152*	0.223	0.351	0.234*
Aae_8252	0.274	0.402	0.452	0.120	0.213	0.239	0.160	0.271	0.234
Aae_8427	0.381	0.477	0.429	0.424	0.494	0.326*	0.309	0.431	0.277*
Aae_8435	0.390	0.482	0.439	0.755	0.374	0.404	0.702	0.423	0.298*
Aae_8734	0.143	0.248	0.286	0.064	0.121	0.128	0.043	0.082	0.085
Aae_9052	0.048	0.092	0.048	0.011	0.021	0.021	0.021	0.042	0.043
Aae_9057	0.256	0.386	0.415	0.287	0.414	0.362	0.298	0.423	0.383
Aae_10384	0.405	0.488	0.571	0.521	0.504	0.447	0.426	0.494	0.553
Aae_10678	0.024	0.048	0.000*	0.032	0.062	0.064	0.000	0.000	0.000
Aae_10801	0.119	0.212	0.238	0.064	0.121	0.128	0.064	0.121	0.128
Aae_11731	0.214	0.341	0.333	0.340	0.454	0.468	0.213	0.339	0.298
Aae_11754	0.060	0.113	0.119	0.032	0.062	0.064	0.000	0.000	0.000
